# Gene Expression Profiling in Peripheral Blood Cells and Synovial Membranes of Patients with Psoriatic Arthritis

**DOI:** 10.1371/journal.pone.0128262

**Published:** 2015-06-18

**Authors:** Marzia Dolcino, Andrea Ottria, Alessandro Barbieri, Giuseppe Patuzzo, Elisa Tinazzi, Giuseppe Argentino, Ruggero Beri, Claudio Lunardi, Antonio Puccetti

**Affiliations:** 1 Institute G. Gaslini, Genova, Italy; 2 University of Verona, Verona, Italy; 3 University of Genova, Genova, Italy; Institute of Immunology, Rikshospitalet, NORWAY

## Abstract

**Background:**

Psoriatic arthritis (PsA) is an inflammatory arthritis whose pathogenesis is poorly understood; it is characterized by bone erosions and new bone formation. The diagnosis of PsA is mainly clinical and diagnostic biomarkers are not yet available. The aim of this work was to clarify some aspects of the disease pathogenesis and to identify specific gene signatures in paired peripheral blood cells (PBC) and synovial biopsies of patients with PsA. Moreover, we tried to identify biomarkers that can be used in clinical practice.

**Methods:**

PBC and synovial biopsies of 10 patients with PsA were used to study gene expression using Affymetrix arrays. The expression values were validated by Q-PCR, FACS analysis and by the detection of soluble mediators.

**Results:**

Synovial biopsies of patients showed a modulation of approximately 200 genes when compared to the biopsies of healthy donors. Among the differentially expressed genes we observed the upregulation of Th17 related genes and of type I interferon (IFN) inducible genes. FACS analysis confirmed the Th17 polarization. Moreover, the synovial trascriptome shows gene clusters (bone remodeling, angiogenesis and inflammation) involved in the pathogenesis of PsA. Interestingly 90 genes are modulated in both compartments (PBC and synovium) suggesting that signature pathways in PBC mirror those of the inflamed synovium. Finally the osteoactivin gene was upregulared in both PBC and synovial biopsies and this finding was confirmed by the detection of high levels of osteoactivin in PsA sera but not in other inflammatory arthritides.

**Conclusions:**

We describe the first analysis of the trancriptome in paired synovial tissue and PBC of patients with PsA. This study strengthens the hypothesis that PsA is of autoimmune origin since the coactivity of IFN and Th17 pathways is typical of autoimmunity. Finally these findings have allowed the identification of a possible disease biomarker, osteoactivin, easily detectable in PsA serum.

## Introduction

Psoriatic arthritis (PsA) is primarily characterised by enthesitis and by synovitis, leading to bone erosions and new bone formation [1]; 10% to 30% of patients with skin psoriasis are affected by the disease, with an estimated prevalence of 1%.

Genetic studies indicate that PsA has a heritable component [2] and many genes have been implicated in psoriasis and PsA [3]. However only a few genes have been associated to both psoriasis and PsA [4].

PsA is characterized by different clinical phenotypes: oligoarticular or polyarticular asymmetrical peripheral joint inflammation or axial involvement. In the last few years several criteria have been used for the classification of PsA. The most frequently classification criteria used are those proposed by Moll and Wright [5] and more recently, are the classification criteria for PsA (CASPAR) [6]. The diagnosis of PsA is mainly performed on a clinical basis and after the exclusion of other seronegative arthritides and up to now there are no diagnostic tests available. Diagnostic work up is based on medical history, physical examination, blood tests, and imaging of the joints. Plain radiographs are used to evaluate the joint damage. Magnetic resonance imaging (MRI) is able to detect joint damage earlier and to assess the extent of joint involvement more accurately than plain radiographs. Indeed MRI is able both to quantify the extent of the inflammatory process within the affected joints and to detect enthesitis even in apparently unaffected joints and in the absence of clinical symptoms. Enthesitis is considered the primary event in the pathogenesis of the disease [7]. Moreover MRI and scintigraphy can be used for an early detection of sacroiliitis and axial disease. In addition these imaging techniques are widely used to evaluate the efficacy of novel therapies for PsA [8,9].

In psoriatic skin lesions the typical cell infiltrate contains activated keratinocytes, T and B lymphocytes, macrophages and neutrophils. Both CD4 and CD8 T cells have been associated with skin and joint damage [10,11] typical of PsA.

The synovial tissue in PsA is characterized by an abundant T cell infiltrate, marked angiogenesis, and synovial hyperplasia with increased expression and/or secretion of cytokines and proteases that contribute to amplify the local inflammation and may explain the “erosive behavior” of the synovium leading to joint destruction.

The cytokine tumor necrosis factor-alpha (TNF-alpha) is a very important inflammatory mediator and has been implicated in PsA pathogenesis. TNF-alpha inhibitors are widely used in PsA therapy and are usually quite effective in reducing the extent of skin lesions and of musculoskeletal symptoms, however a high percentage of PsA patients does not respond to TNFalpha antagonists. Considering the expense and side effects of anti-TNF biological agents, the identification of biomarkers that could be used to predict which patients will respond to biological treatment is an important goal in medicine.

Therapies that target the TNFα induce a significant clinical improvement in approximately 70% of patients [12]. However, the extent of clinical improvement is often far from complete remission and the majority of PsA patients experience a flare of the disease within the first 2 years [12]. Therefore, the identification of new molecules that play a pivotal role in the pathogenesis of the disease is fundamental for the development of new therapies. Moreover prognostic and diagnostic biomarkers need to be identified. To this aim, gene expression profiling may be of great help since it has been used to classify lymphoid malignancies [13] and to dissect pathways involved in several autoimmune and inflammatory diseases. In this regard gene array studies have generated useful insight in several diseases including systemic lupus erythematosus (SLE) [14], rheumatoid arthritis (RA) [15], and multiple sclerosis (MS) [16]

Gene expression studies in PBMC of subjects with rheumatoid arthritis (RA) and systemic lupus erythematosus (SLE) have allowed the identification of specific gene 'signatures' that are differentially expressed in patients when compared to healthy subjects [14,15]. In a few cases differentially expressed genes (DEGs) have been shown to correlate with particular features of the disease (such as presence or absence of systemic organ involvement in RA), or with capacity to respond to immunosuppressive treatment.

In this study we used a gene array strategy to identify transcriptional profiles that distinguish PsA patients from healthy control subjects. For this purpose we analyzed for the first time gene expression in paired peripheral blood cells (PBC) and synovial biopsies of 10 PsA patients. We reasoned that the combined analysis of transcription data obtained in PBC and in the target tissue of PsA, the synovial membrane, could provide a deeper understanding of the genetic pathways that regulate the disease.

Using this approach we have been able to shed light on some aspects of disease pathogenesis by dissecting different aspects of this complex pathology. Interestingly DEGs recapitulate most of the typical features of PsA, such as bone remodeling with new bone formation, synovial hyperplastic growth with neoangiogenesis, local and systemic inflammatory response, activation of innate and adaptive immune reponses.

Moreover our results provide evidence for an autoimmune origin of PsA, since gene expression profiles are characterized by the combined overexpression of type I IFN inducible genes [17] and of Th17 related transcripts. Indeed coactivity of IFN and Th17 pathways is typical of autoimmunity and has been described both in human diseases and in animal models [18–20].

Moreover, using the same strategy, we could identify a novel biomarker, osteoactivin, that can be easily detected in PsA sera. Indeed increased serum levels of osteoactivin, seem to be typical of PsA and, in the absence of other disease markers, the detection of high levels of this molecule may represent an interesting tool in the diagnostic work up of PsA.

## Patients and Methods

### Patients

We studied a cohort of 60 patients (38 males and 22 females, mean age: 44 years) affected by PsA, attending the Unit of Autoimmune Diseases, at the University Hospital of Verona, Italy. The patients enrolled in the study were selected as already described elsewhere [21]. All patients fulfilled the CASPAR criteria for the diagnosis of PsA: inflammatory musculoskeletal involvement (inflammatory arthritis, enthesitis or lumbar pain) combined with at least 3 features: 1) evidence of current psoriasis, personal history of psoriasis, family history of psoriasis in unaffected patiens; 2) affected nails (onycholysis, pitting); 3) dactylitis; 4) negative rheumatoid factor; 5) radiographic evidence of new juxta-articular bone formation (excluding osteophytes) [22].

All the patients underwent clinical examination and laboratory evaluation comprehensive of inflammatory markers, such as CRP and erythrocytes sedimentation rate (ESR); rheumatoid factor (RF) and anti-CCP antibody detected by ELISA test; antinuclear antibody detected by indirect immunofluorescence on HEp-2 cells; and genetic screening for the association with the allele HLA-B27. All patients underwent the following instrumental investigations: ultrasonography with Power Doppler to investigate subclinical enthesopaty and synovitis in asymptomatic patients; conventional radiography, magnetic resonance imaging (MRI) and scintigraphy. The radiological features of peripheral PsA included asymmetric distribution, participation of distal interphalangeal joints, periostitis, bone density preservation, bone ankylosis and pencil-in-cup deformity.

A group of 10 subjects with recent onset PsA (within one year from the diagnosis) was selected within the entire cohort of PsA patients and utilized for the gene array study. These patients were not treated with anti-TNF agents or with disease-modifying antirheumatic drugs (DMARDs). The clinical features of the patients are reported in Table 1 that also includes a description of the PsA patients selected for the gene array study. Sixty control subjects matched for sex and age served as control group. Moreover 60 patients with rheumatoid arthritis (RA), 60 patients with Ankylosing spondylitis (AS) and 20 patients with osteoarthritis (OA) were used as controls. RA patients had the American College of Rheumatology classification criteria for RA [23]. In all RA patients the disease was at an active stage and blood was obtained before therapy with biological agents. AS patients met the SAS classification criteria for AS [24].

**Table 1:** Clinical features of the patients with PsA included in the study.

**Patients**		**60**
Sex	male	38
	female	22
Age at diagnosis (years)		44±8
Involvement	axial	25
	peripheral	35
Enthesitis		29
Dactylitis		23
Psoriasis		47
Association with HLA-B27		18
**Patients utilized for the gene array study**		**10**
Sex	male	6
	female	4
Age at diagnosis (years)		43±6
Involvement	axial	4
	peripheral	6
Enthesitis		7
Dactylitis		4
Psoriasis		7
Association with HLA-B27		3

All the participants to the study signed a written informed consent. The local Ethical Committee of the Azienda Ospedaliera Universitaria of Verona, Verona, Italy had approved the study protocol. All the investigations have been performed according to the principles contained in the Helsinki declaration.

### Synovial biopsies

In the 10 PsA patients selected for the gene array study synovial membranes were obtained with needle biopsies.

In 10 control healthy donors synovial biopsies were collected during orthopedic knee arthroscopy following traumatic events.

### Gene array

Tissue samples from every single patient were frozen in liquid nitrogen immediately after dissection and stored at -70°C until homogenization. Sample preparation for gene array analysis was carried out as previously described [25–27]. Briefly, frozen samples were homogenized in TRI REAGENT (1mL per 50–100mg of tissue) in a Potter-type mechanical homogenizer with Teflon pestle.

PAXgene Blood RNA tubes (PreAnalytiX, Hombrechtikon, Switzerland) were used for blood collection and total RNA was extracted according to the protocol supplied by the manufacturer.

Preparation of cRNA hybridization and scanning of arrays for each samples were performed following the manufacturer instructions (Affymetrix, Santa Clara, CA, USA) by Cogentech Affymetrix microarray unit (Campus IFOM IEO, Milan, Italy) using the Human Genome U133A 2.0 Gene Chip (Affymetrix). The Human Genome U133A Gene Chip is a single array which contains 14,500 well-characterized human genes and more than 22,000 probe sets and 500,000 distinct oligonucleotide features. The gene expression profiles were analyzed using the Gene Spring software, version 12.1 (Agilent Technologies, SantaClara, CA, USA) that calculated a robust multi-array average of background-adjusted, normalized, and log-transformed intensity values applying the Robust Multi-Array Average algorithm (RMA).

With this software the mean optical background level for each array was subtracted from the signal intensity for each probe. The normalized data were transformed to the log2 scale. A signal log2 ratio of 1.0 corresponds to an increase of the transcript level by two-fold change (2 FC) and -1.0 indicates a decrease by two-fold change (-2 FC). A signal log2 ratio of zero would indicate no change. The unpaired t-test was performed to determine which genes were modulated at a significant level (p≤0.01) and p values were corrected for multiple testing by using Bonferroni correction. Finally, statistically significant genes were chosen for final consideration when their expression was at least 2.0 fold different in the test sample versus control sample. Genes that passed both the p-value and the FC restriction were submitted to functional classification analysis according to the Gene Ontology (GO) annotations.

The microarray results have been reported according to the MIAME guidelines and deposited in the public repository ArrayExpress http://www.ebi.ac.uk/arrayexpress; accession number E-MTAB-3201.

### FACS analysis

Peripheral blood mononuclear cells (PBMCs) were obtained from 20 healthy donors and 30 patients affected by PsA following a density-gradient centrifugation on Lymphoprep (Nycomed Pharma, Oslo, NO) and two washes with PBS.

Cells collected from patients and normal controls were cultured in 2 mL tubes containing 1 mL of RPMI 1640 + FCS 10% (Lonza, Basel, CH) at a concentration of 1*10^6^ cells/mL. Cells were stimulated for 4 hours with Dynabeads Human T-Activator CD3/CD28 (Life Technologies, Carlsbad, CA, USA) or with heat-inactivated *Candida albicans*. IL-17 production was assessed using the IL-17 Secretion Assay (Miltenyi Biotec, Bergish Gladbach, D) according to the manufacturer’s instruction. Briefly, cells were washed in 2 mL of cold buffer, centrifuged at 300xg for 5 minutes at 4°C and the pellet resuspended in 90 μL of cold medium. An incubation with IL-17 Catch Reagent was carried out for 5 minutes in ice and cells were washed as before. PBMCs were incubated in 1 mL of warm medium at 37°C for 45 minutes under slow continuous rotation. Cells were then washed and resuspended in in 75 μL of cold buffer and 10 μL of IL-17 Detection Antibody APC, 10 μL of anti-CD3 PerCP (Becton Dickinson, Franklin Lakes, NJ, USA) and 5 μL of anti-CD4 APC-H7 (Becton Dickinson) antibodies were added. Incubation was performed in ice for 10 minutes. Finally cells were washed and resuspended in an appropriate volume of PBS and acquired on a FACSCanto II cytometer (Becton Dickinson). Analysis was performed with FlowJo 9.3.3 software (Tree Star, Ashland, OR, USA).

### Real Time RT-PCR

Total RNA was isolated from PBMC using TRIzol reagent (Invitrogen, Carlsbad, CA, USA), following manufacturer’s instructions. First-strand cDNA was generated using the SuperScript III First-Strand Synthesis System for RT-PCR Kit (Invitrogen), with random hexamers, according to the manufacturer’s protocol. PCR was performed in a total volume of 25 μl containing 1× Taqman Universal PCR Master mix, no AmpErase UNG and 2.5 μl of cDNA; pre-designed, Gene-specific primers and probe sets for each gene (SPP1: Hs 00959010_m1; CXCL13: Hs 00757930_m1; CCL18 Hs 00268113_m1; LAMP3: Hs 00180880_m1; CADM1: Hs 00942509_m1) were obtained from Assay-on-Demande Gene Expression Products (Applied Biosystems).

As described in details previously [25–27], Real Time PCR reactions were carried out in a two-tube system and in singleplex. The Real Time amplifications included 10 minutes at 95°C (AmpliTaq Gold activation), followed by 40 cycles at 95°C for 15 seconds and at 60°C for one minute. Thermocycling and signal detection were performed with 7500 Sequence Detector (Applied Biosystems). Signals were detected according to the manufacturer’s instructions. This technique allows the identification of the cycling point where PCR product is detectable by means of fluorescence emission (Threshold cycle or Ct value). As previously reported, the Ct value correlates to the quantity of target mRNA [26]. Relative expression levels were calculated for each sample after normalization against the housekeeping genes GAPDH, beta-actin and 18s ribosomal RNA (rRNA), using the ΔΔCt method for comparing relative fold expression differences [25,28]. The data are expressed as fold change. Ct values for each reaction were determined using TaqMan SDS analysis software. For each amount of RNA tested triplicate Ct values were averaged. Because Ct values vary linearly with the logarithm of the amount of RNA, this average represents a geometric mean.

### Detection of soluble mediators in sera and synovial fluids

Serum levels of osteoactivin/GPNMB [29] were detected using a commercially available ELISA kit (Biorbyt Ltd, Cambridge, UK). The ELISA kits for osteopontin, CCL20, CCL18, MMP-3 and IL-23 were all purchased from R & D Systems (Phoenix, AZ, USA) and used according to the manufacturer’s instructions. IL-17 and IL-23 were detected in synovial fluids (SFs) of 20 patients with PsA and in 20 patients with OA, used as controls. The ELISA kit for IL-17 was purchased from eBioscience (San Diego, CA, USA) and used according to the manufacturer’s instruction.

SF was distributed into heparinized tubes, and the cells removed by centrifugation (400 × *g*, 10 min). Before each test. synovial fluids were treated with hyaluronidase at a concentration of 10 U/ml for 80 min at 37°C followed by centrifugation (12,000 × *g*, 5 min). This treatment was used to reduce the viscosity of synovial fluid.

### Statistical Analysis

Statistical testing was performed using SPSS Statistics 2 software (IBM,United States). Data obtained from the analysis of the soluble mediators were submitted to statistical testing using the non parametric Mann-Whitney test. For the analysis of IL-17–positive CD4+T cells in PBMCs and of IL-17 and IL-23 synovial fluid levels, the significance of the differences between patients and controls was determined using the unpaired Student’s t-test.

## Results

### Gene-array analysis

#### a) DEGs in synovial biopsies

In order to identify genes potentially involved in the pathogenesis of PsA, we first compared the gene expression profiles of 10 synovial biopsies obtained from patients with PsA with synovial specimens obtained from normal healthy subjects undergoing post-traumatic surgery.

When both a Bonferroni–corrected *P*-value criterion (p≤0.01) and a fold change criterion (FC≥2) were applied to the signal variation of every single gene to detect robust and statistically significant changes between baseline and experimental arrays [25,27], we obtained 196 modulated genes that were further analyzed. 135 and 61 transcripts resulted to be up- and downregulated respectively; in many cases the fold changes were very high. In particular three genes showed a very high level of induction: osteopontin, SPP1/OPN (FC 450), fibronectin 1, FN1 (FC 405) and osteoactivin, GPNMB (FC 147).

**Fig 1:**
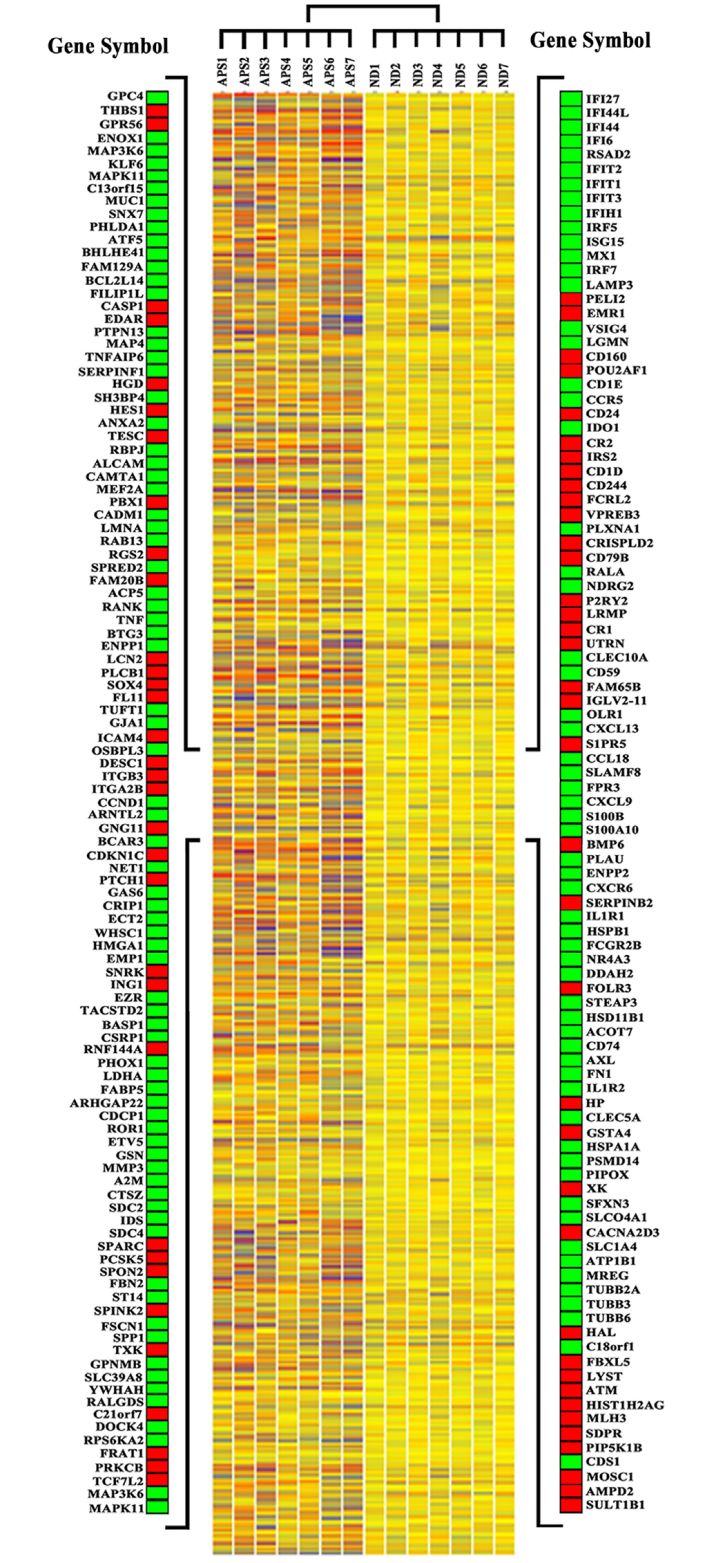
Gene expression profiles of synovial membranes obtained from 10 PsA patients and from 10 healthy subjects. The heat map shows the expression levels of all the modulated genes. Blue-violet indicates genes that are expressed in synovial biopsies of PsA patients at lower levels when compared with the mean values of the control subjects, orange-red indicates genes that are expressed at higher levels when compared to the control means and yellow indicates genes that are not differently expressed in the patients group versus the control group. Each row represents a gene; each column shows the expression of selected genes in each individual (see S1 Table for complete list of genes expression data). On both sides of the heat map a selection of genes is shown. Green and red squares indicate upregulated and downregulated genes respectively.

Fig 1 shows the heat map representing some Affymetrix arrays with a selection of modulated genes.

The Gene Ontology analysis of the regulated transcripts showed that the vast majority of them are involved in several biological processes that may play a role in PsA including: inflammation, immune response, apoptosis, cell cycle regulation and proliferation, cell migration and invasion, extra-cellular matrix (ECM) and ECM remodeling, bone remodeling, angiogenesis, signal transduction.

Table 2 shows a detailed representation of selected genes within the above-mentioned clusters. The table also includes GeneBank accession numbers and fold changes. The complete list of modulated genes can be found in S1 Table.

**Table 2:** Annotated genes differentially expressed in PsA synovial membranes versus healthy synovial membranes grouped according to their function.

Probe Set ID	Gene Title	Gene Symbol	FC	Representative Public ID
**inflammation**				
207113_s_at	tumor necrosis factor (TNF superfamily, member 2)	TNF	3.55	NM_000594
210004_at	oxidized low density lipoprotein (lectin-like) receptor 1	OLR1	83.10	AF035776
205242_at	chemokine (C-X-C motif) ligand 13	CXCL13	79.22	NM_006419
32128_at	chemokine (C-C motif) ligand 18	CCL18	20.32	NM_002988
203915_at	chemokine (C-X-C motif) ligand 9	CXCL9/MIG	11.16	NM_002416
209686_at	S100 calcium binding protein B	S100B	7.80	NM_006272
200872_at	S100 calcium binding protein A10	S100A10	2.21	NM_002966
206974_at	chemokine (C-X-C motif) receptor 6	CXCR6	6.28	NM_006564
202948_at	interleukin 1 receptor, type I	IL1R1	5.67	NM_000877
211719_x_at	fibronectin 1	FN1	405.39	NM_212482
**immune response**				
**a) adaptive**				
205242_at	chemokine (C-X-C motif) ligand 13	CXCL13	79.22	NM_006419
216876_s_at	interleukin 17A	IL17A	2.30	U32659
221165_s_at	interleukin 22	IL22	2.04	AF279437
221111_at	interleukin 26	IL26	3.67	NM_018402
220054_at	interleukin 23, alpha subunit p19	IL23A	2.00	NM_016584
208991_at	signal transducer and activator of transcription 3	STAT3	2.26	NM_139276.2
206983_at	chemokine (C-C motif) receptor 6	CCR6	2.01	NM_004367
205476_at	chemokine (C-C motif) ligand 20	CCL20	3.61	NM_004591
206991_s_at	chemokine (C-C motif) receptor 5	CCR5	8.42	NM_000579
208488_s_at	complement component (3b/4b) receptor 1	CR1	-2.36	NM_000651
205544_s_at	complement component receptor 2	CR2	-5.05	NM_001877
221239_s_at	Fc receptor-like 2	FCRL2	-3.90	NM_030764
220068_at	pre-B lymphocyte 3	VPREB3	-3.59	NM_013378
**b) innate**				
205569_at	lysosomal-associated membrane protein 3	LAMP3	36.99	NM_014398
204787_at	V-set and immunoglobulin domain containing 4	VSIG4/Z39IG	26.76	NM_007268
221538_s_at	plexin A1	PLXNA1	3.36	NM_032242
210889_s_at	Fc fragment of IgG, low affinity IIb, receptor (CD32)	FCGR2B	5.25	M31933
209959_at	nuclear receptor subfamily 4, group A, member 3	NR4A3	5.20	U12767
219890_at	C-type lectin domain family 5, member A	CLEC5A	16.91	NM_013252
206682_at	C-type lectin domain family 10, member A	CLEC10A	4.01	NM_006344
**apoptosis**				
205573_s_at	sorting nexin 7	SNX7	7.76	NM_015976
217996_at	pleckstrin homology-like domain, family A, member 1	PHLDA1	6.09	NM_007350
204998_s_at	activating transcription factor 5	ATF5	5.79	NM_012068
221530_s_at	basic helix-loop-helix family, member e41	BHLHE41	5.03	AB044088
217966_s_at	family with sequence similarity 129, member A	FAM129A	3.54	AF288391
211367_s_at	caspase 1, apoptosis-related cysteine peptidase	CASP1	-2.83	NM_033292
220048_at	ectodysplasin A receptor	EDAR	-2.22	NM_022336
204201_s_at	protein tyrosine phosphatase, non-receptor type 13	PTPN13	2.18	NM_006264
243_g_at	microtubule-associated protein 4	MAP4	2.40	M64571
204614_at	serpin peptidase inhibitor, clade B, member 2	SERPINB2	-5.90	NM_002575
**cell cycle regulation**				
208712_at	cyclin D1	CCND1	9.94	NM_053056.2
220658_s_at	aryl hydrocarbon receptor nuclear translocator-like 2	ARNTL2	4.03	NM_020183
204115_at	guanine nucleotide binding protein (G protein), gamma 11	GNG11	-42.28	NM_004126
204032_at	breast cancer anti-estrogen resistance 3	BCAR3	3.94	NM_003567
**cell proliferation**				
213348_at	cyclin-dependent kinase inhibitor 1C (p57, Kip2)	CDKN1C	-17.49	NM_001122631
201830_s_at	neuroepithelial cell transforming 1	NET1	4.27	NM_005863
209815_at	patched homolog 1 (Drosophila)	PTCH1	-7.22	NM_000264
202177_at	growth arrest-specific 6	GAS6	4.12	NM_000820
205081_at	cysteine-rich protein 1	CRIP1	3.11	NM_001311
219787_s_at	epithelial cell transforming sequence 2 oncogene	ECT2	3.04	NM_018098
201324_at	epithelial membrane protein 1	EMP1	45.31	NM_001423
209481_at	SNF related kinase	SNRK	-2.15	AF226044
202286_s_at	tumor-associated calcium signal transducer 2	TACSTD2	56.82	NM_002353
**cell migration and/or invasion**				
202345_s_at	fatty acid binding protein 5 (psoriasis-associated)	FABP5	12.73	NM_001444
218451_at	CUB domain containing protein 1	CDCP1	3.61	NM_022842
203349_s_at	ets variant 5	ETV5	7.03	NM_004454
200696_s_at	gelsolin (amyloidosis, Finnish type)	GSN	3.65	NM_000177
**ECM/ECM remodeling**				
205828_at	matrix metallopeptidase 3 (stromelysin 1, progelatinase)	MMP3	26.05	NM_002422
217757_at	alpha-2-macroglobulin	A2M	25.43	NM_000014
210042_s_at	cathepsin Z	CTSZ	7.14	NM_001336
212158_at	syndecan 2	SDC2	15.68	NM_002998
202439_s_at	iduronate 2-sulfatase	IDS	2.31	NM_000202
202071_at	syndecan 4	SDC4	5.98	NM_002999
205559_s_at	proprotein convertase subtilisin/kexin type 5	PCSK5	-4.52	NM_006200
206310_at	serine peptidase inhibitor, Kazal type 2	SPINK2	-2.30	NM_021114
214768_x_at	anti-thyroid peroxidase monoclonal autoantibody IgK chain, V region	FAM20B	-4.03	NM_014864
**angiogenesis**				
204984_at	glypican 4	GPC4	2.24	NM_001448
201110_s_at	thrombospondin 1	THBS1	-8.29	NM_003246
212070_at	G protein-coupled receptor 56	GPR56	-7.31	NM_001145774
219501_at	ecto-NOX disulfide-thiol exchanger 1	ENOX1	3.47	NM_017993
219278_at	mitogen-activated protein kinase kinase kinase 6	MAP3K6	2.41	NM_004672
211499_s_at	mitogen-activated protein kinase 11	MAPK11	2.24	NM_002751
213693_s_at	mucin 1, cell surface associated	MUC1	2.46	X80761
**bone remodeling**				
209875_s_at	secreted phosphoprotein 1	SPP1/OPN	448.78	J04765
201141_at	glycoprotein (transmembrane) nmb	GPNMB	147.53	NM_001005340
**a) bone growth**				
209031_at	cell adhesion molecule 1	CADM1	14.85	NM_014333
203411_s_at	lamin A/C	LMNA	13.75	NM_005572
212458_at	sprouty-related, EVH1 domain containing 2	SPRED2	4.33	AY299090
205066_s_at	ectonucleotide pyrophosphatase/phosphodiesterase 1	ENPP1	7.20	NM_006208
206026_s_at	tumor necrosis factor, alpha-induced protein 6	TNFAIP6	4.84	NM_007115
202283_at	serpin peptidase inhibitor, clade F, member 1	SERPINF1	4.77	NM_002615
201951_at	activated leukocyte cell adhesion molecule	ALCAM	5.95	L38608
**b) bone erosion**				
207113_s_at	tumor necrosis factor (TNF superfamily, member 2)	TNF	3.55	NM_000594
204638_at	acid phosphatase 5, tartrate resistant	ACP5	3.91	NM_001611
207037_at	tumor necrosis factor receptor superfamily, member 11a, NFKB activator	TNFRSF11A	3.77	NM_003839
202252_at	RAB13, member RAS oncogene family	RAB13	5.37	NM_002870
212151_at	pre-B-cell leukemia homeobox 1	PBX1	-2.36	NM_002585
202388_at	regulator of G-protein signaling 2, 24kDa	RGS2	-4.34	NM_002923
212531_at	lipocalin 2	LCN2	-3.26	NM_005564
201417_at	SRY (sex determining region Y)-box 4	SOX4	-2.78	NM_003107
203395_s_at	hairy and enhancer of split 1	HES1	-2.18	NM_005524
**signaling pathways**				
**a) Wnt and beta-catenin pathway**				
205003_at	dedicator of cytokinesis 4	DOCK4	13.81	AY233380
213693_s_at	mucin 1, cell surface associated	MUC1	2.46	X80761
219889_at	frequently rearranged in advanced T-cell lymphomas	FRAT1	-2.59	NM_005479
205805_s_at	receptor tyrosine kinase-like orphan receptor 1	ROR1	2.02	NM_005012
208712_at	cyclin D1	CCND1	9.94	NM_053056.2
201667_at	gap junction protein, alpha 1, 43kDa	GJA1	9.03	NM_000165
216511_s_at	transcription factor 4	TCF4/TCF7L2	-7.27	NM_001146274
**b) MAP-kinase pathway**				
219278_at	mitogen-activated protein kinase kinase kinase 6	MAP3K6	2.41	NM_004672
211499_s_at	mitogen-activated protein kinase 11	MAPK11	2.24	NM_002751
212912_at	ribosomal protein S6 kinase, 90kDa, polypeptide 2	RPS6KA2	2.65	NM_021135
202581_at	heat shock 70kDa protein 1A	HSPA1A	6.94	NM_005345
**d) Insulin pathway**				
209185_s_at	insulin receptor substrate 2	IRS2	-4.83	AF073310
205066_s_at	ectonucleotide pyrophosphatase/phosphodiesterase 1	ENPP1	7.20	NM_006208
206020_at	suppressor of cytokine signaling 6	SOCS6	2.26	NM_004232
**b) Notch pathway**				
211974_x_at	recombination signal binding protein for immunoglobulin kappa J region	RBPJ	2.92	NM_203284
203395_s_at	hairy and enhancer of split 1	HES1	-2.18	NM_005524
207113_s_at	tumor necrosis factor (TNF superfamily, member 2)	TNF	3.55	NM_000594
**e) Type I interferon pathway**				
202411_at	interferon, alpha-inducible protein 27	IFI27	13.74	NM_005532
204439_at	interferon-induced protein 44-like	IFI44L	2.23	NM_006820
214453_s_at	interferon-induced protein 44	IFI44	2.32	NM_006417
204415_at	interferon, alpha-inducible protein 6	IFI6	2.20	NM_022873
213797_at	radical S-adenosyl methionine domain containing 2	RSAD2	3.68	NM_080657
217502_at	interferon-induced protein with tetratricopeptide repeats 2	IFIT2	2.03	NM_001547
203153_at	interferon-induced protein with tetratricopeptide repeats 1	IFIT1	3.17	NM_001548
204747_at	interferon-induced protein with tetratricopeptide repeats 3	IFIT3	2.58	NM_001549
219209_at	interferon induced with helicase C domain 1	IFIH1	2.15	NM_022168
205469_s_at	interferon regulatory factor 5	IRF5	2.03	NM_001098629
205483_s_at	ISG15 ubiquitin-like modifier	ISG15	2.03	NM_005101
202086_at	myxovirus resistance 1, interferon-inducible protein p78	MX1	3.05	NM_002462
208436_s_at	interferon regulatory factor 7	IRF7	2.15	NM_001572
**f) others**				
201020_at	tyrosine 3-monooxygenase/tryptophan 5-monooxygenase activation protein, eta	YWHAH	3.27	NM_003405
209050_s_at	ral guanine nucleotide dissociation stimulator	RALGDS	2.43	NM_006266
221211_s_at	chromosome 21 open reading frame 7	C21orf7	-8.00	NM_020152

A large number (29/196) of modulated genes have a role in the inflammatory process.

The upregulated transcripts comprise chemokine (C-X-C motif) ligand 13, CXCL13 (FC 79.22), CXCL9/MIG (FC 11.16), chemokine (C-C motif) ligand 18, CCL18 (FC 20.32), interleukin 1 receptor, type I, IL1R1 [30,31] (FC 5.67), oxidized low density lipoprotein receptor 1, OLR1/LOX1 (FC 83.1), S100 calcium binding protein namely S100B [32] (FC 7.8) and S100A102 [33] (FC 2.21), fibronectin1, FN1 [34] (FC 405.4), tumour necrosis factor alpha TNF (FC 3.5).

Among genes involved in the immune response, many Th17-lymphocytes related genes were up-regulated including CCR6 (FC 2.01), CCL20 (FC 3.6), CXCL13 (FC 79.2) [35] interleukin 17A, IL17A [36] (FC 2.3), IL22 [37] (FC 2.04), IL26 [38] (FC 3.67); IL23A [39] (FC 2.2) and signal transducer and activator of transcription 3, STAT3 (FC 2.26) [40].

Other genes involved in B cell activity are downregulated in PsA samples [40,41].

Several upregulated genes play a role in innate immunity and are expressed in dendritic cells [42–45] and in macrophages [46].

Many genes coding for protein involved in apoptosis and/or in apoptosis regulation resulted modulated in pathological samples [47–54].

Genes that positively regulate cell cycle resulted overexpressed in PsA synovial samples [55–57]. Moreover there was a strong downregulation of the guanine nucleotide binding protein, gamma 11, GNG11 (FC -42.3), an inducer of cellular senescence in human cells [58]. Genes that control cell proliferation were also modulated in PsA biopsies even with very high fold change [59–66].

Another group of differentially expressed genes comprises transcripts involved in cell migration and invasion. Interestingly genes that promote these processes resulted up-regulated, i.e. the CUB domain containing protein 1, CDCP1 (FC 3.6) [67]; gelsolin, GSN (FC 3.65) [68]; the fatty acid binding protein 5, FABP5 (FC 12.73) [69], and the ets variant 5, ETV5 (FC 7.03), a pro-invasive transcription factor tought to be involved in bone invasion [70].

Several genes encoding for ECM components or involved in ECM remodeling were modulated in PsA samples. We found a strong overexpression of syndecan 2, SDC2 (FC 15.68) and syndecan 4, SDC4 (FC 5.98) and of several proteases-encoding genes, including matrix metallopeptidase 3, MMP3 (FC 26.05), suppression of tumorigenicity 14, ST14/matriptase (FC 2.78); carboxy-peptidase cathepsin Z, CTSZ (FC 7.14) [71] and iduronate 2-sulfatase, IDS (FC 2.31) [72]. On the contrary, inhibitors of metalloproteases, proprotein convertase subtilisin/kexin type 5, PCSK5/PC5/6 (FC -4.52) [73], and serine peptidase inhibitor, Kazal type 2, SPINK2 (FC -2.30) [74] were underexpressed.

Differentially expressed genes also comprise transcripts which regulate the angiogenesis process [75–78].

A large number (28/196) of genes differentially expressed in PsA biopsies belong to the bone remodeling cluster which comprises transcripts involved in bone-growth and bone resorption. Upregulated genes promote bone growth with different mechanisms such as osteoblast differentiation i.e: lamin A, LMNA (FC 13.75) [79]; bone-matrix deposition, i.e: cell adhesion molecule 1, CADM1 (FC 14.85) [80]; bone development, i.e: sprouty-related, EVH1 domain containing 2, SPRED2 (FC 4.33) [81]; bone mineralization i.e: ectonucleotide pyrophosphatase/phosphodiesterase 1, ENPP1/NPP1 (FC 7.20), [82] and bone morphogenesis, i.e: activated leukocyte cell adhesion molecule, ALCAM/CD166 (FC 5.95), [83].

Genes inhibiting osteoblast proliferation [84,85]and osteoblast differentiation [86–88] were expressed in lower levels in PsA samples.

In addition the following upregulated genes may support bone growth by interfering with osteoclast activity: tumor necrosis factor, alpha-induced protein 6, TNFAIP6/TSG6 (FC 4.84) [89], and serpin peptidase inhibitor, clade F member 1, SERPINF1/PEDF (FC 4.77).

In the bone resorption cluster, the following upregulated genes promote osteoclast activity and/or differentiation: the acid phosphatase 5, tartrate resistant, ACP5/TRAP (FC 3.91) [90], the tumor necrosis factor a, TNFa (FC 3.55); member RAS oncogene family, RAB13 (FC 5.37) [91] and tumor necrosis factor receptor superfamily, member 11a, NFKB activator, TNFRSF11A/RANK (FC 3.77) [92].

Noteworthy the bone remodeling category contains two genes, osteopontin and osteoactivin, which show a very high level of induction (FC 450 and 147 respectively).

Finally we analysed DEGs which belong to /or are connected to the gene cluster signal transduction using a pathway analysis tool to evaluate if they contribute to known signaling networks that may be relevant in the pathogenesis of PsA and found that a conspicuous number of genes (also including genes originally ascribed to different functional classes) is involved in well characterized signaling networks already associated to human diseases, including immune-mediated diseases, bone diseases and inflammatory diseases.

These signal cascades include: 1) the Interferon-alpha (IFN-A) pathway also named “Type I Interferon signature” [93], 2) the Wnt/beta-catenin signaling pathway, 3) the Notch signaling pathway, 4) the mitogen activated protein (MAP) kinase pathway, and 5) the insulin pathway.

In particular 13 type I interferon inducible genes (IFIG) were upregulated, thus indicating the presence of an IFN type I signature, typically associated with autoimmune disease such as systemic lupus erythematosus (SLE), rheumatoid arthritis (RA), Crohn’s disease and Sjogren syndrome [94–100].

Seven modulated genes belong to the WNT signalling pathway which has been associated with the pathogenesis of RA, and in particular with synovial inflammation and bone remodeling [101–107].

Three genes belong to the Notch signalling pathway that is crucial for bone homeostasis and is involved in the pathogenesis of several immune-mediated diseases, including RA [108].

Four genes of the MAP kinase pathway are upregulated including: mitogen-activated protein kinase 11, MAPK11 (FC 2.24), mitogen-activated protein kinase 6,MAP3K6 (FC 2.41), ribosomal protein S6 kinase, 90 kDa, polypeptide 2, RPS6KA2 (FC 2.65), heat shock 70 kDa protein 1 A, HSPA1A (FC 6.94). The p38 MAPK signalling pathway has been associated with psoriasis and psoriatic arthritis [109].

Two genes belong to the insulin pathway: insulin receptor substrate 2, IRS2 (FC-4.83) and ectonucleotyde pyrophosphatase/phosphodiesterase 1, ENPP1 (FC 7.2). The insulin pathway is involved in the pathogenesis of the metabolic syndrome [110] and an increased body mass index increases the risk of PsA development in patients with cutaneous psoriasis, supporting a link between fat-mediated inflammation and joint involvement [111].

**Fig 2:**
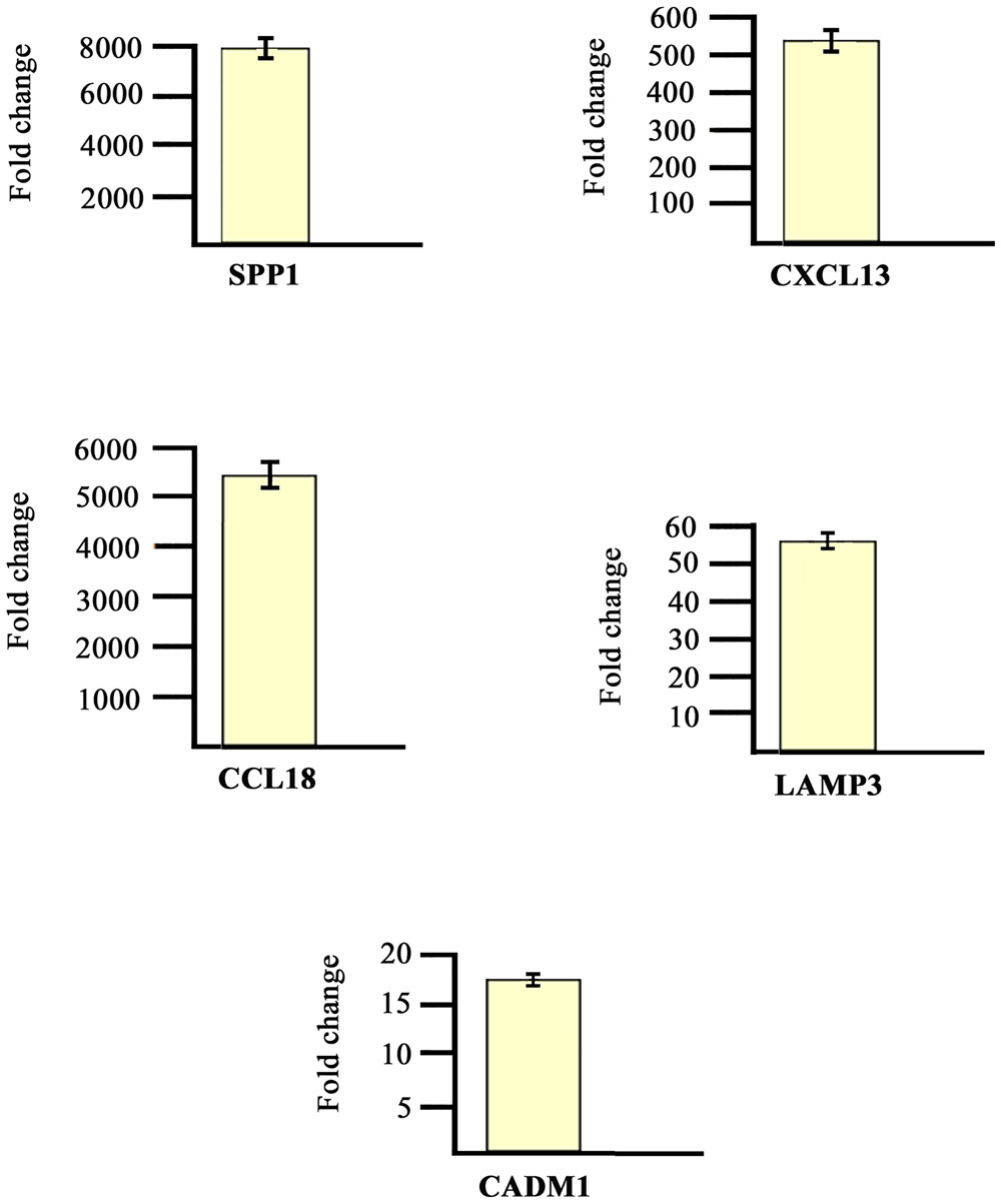
Real time RT-PCR of some modulated genes. Genes selected for validation were SPP1, CXCL13, CCL18, LAMP3 and CADM1. The transcripts of the selected genes were increased in PsA samples when compared to healthy donors. Relative expression levels were calculated for each sample after normalization against the housekeeping gene GAPDH. Experiments have been conducted in triplicates. Similar results were obtained using the housekeeping genes18s rRNA and beta-actin (data not shown).

The modulation of some genes observed by gene array analysis was validated by Q-PCR (Fig 2).

#### B) DEGs in PBC

We next compared the gene expression profiles of 10 PBC samples obtained from the same PsA patients used for the biopsy collection with 10 PBC samples obtained from healthy age and sex matched donors. When the same statistical criteria and fold change criteria were applied to the modulated genes, we obtained 187 genes that were further analysed. 118 and 69 genes resulted to be up- or downregulated respectively. While the number of DEGs in PBC is similar to that obtained in synovial biopsies, the average level of gene induction (FC) is much lower in PBC when compared to the synovium. DEGs are distributed in several functional classes which partially overlap with the ones identified by genes modulated in PsA synovium. The gene categories comprise: inflammation, immune response, apoptosis, cell cycle regulation and proliferation, cell migration and invasion, ECM/ECM remodeling, bone remodelling, angiogenesis, signal transduction.

A detailed representation of modulated genes within the above-mentioned clusters vcan be found in S2 Table.

Interestingly modulated genes are distributed in various gene categories that regulate different biological processes (ie proliferation, apoptosis, immune response), however the functional classes which show the highest enrichment in modulated trascripts are the immune response (36/187), the signal transduction (25/187) the inflammation (25/187), and the bone remodeling (17/187) gene clusters.

Remarkably, in the immune response group, we observed increased expression of several Th17 related genes (CCR6 [112]; CCL20 [113]; lymphocyte antigen 9, LY9 [114,115]; interleukin 6 signal transducer, IL6ST [116–118], interleukin 12 receptor, beta 2, IL12RB [119]).

Noteworthy 10 IFIG were modulated also in PBC samples, thus confirming the presence of an INFA signature observed within the synovium.

Consistently with the presence of a strong inflammatory response typical of PsA we also observed upregulation of several proinflammatory genes (IL8; CCL18; chemokine (C-X-C motif) ligand 1, CXCL1; IL1A; OLR1; HSPA1A; CXCL9; see S2 Table). New bone formation is a typical feature of PsA and consistently with this aspect of the disease several genes involved in the bone growth process are modulated also in PBC.

The analysis of the expression profiles of the PBC samples revealed that 90 out of 187 transcripts differently regulated in PBC were also modulated in PsA synovium (Table 3). These genes belong to different functional classes including: immune response and inflammation; angiogenesis, apoptosis; bone remodeling; cell proliferation; extracellular matrix remodeling and ECM components; signal transduction.

**Table 3:** Annotated genes differentially expressed in PsA synovial membranes and in PsA PBC versus healthy controls grouped according to their function.

Probe Set ID	Gene Title	Gene Symbol	FC SM	FC PBC	Representative Public ID
**inflammation**					
210004_at	oxidized low density lipoprotein (lectin-like) receptor 1	OLR1	83.10	7.16	AF035776
32128_at	chemokine (C-C motif) ligand 18	CCL18	20.32	2.03	NM_002988
219386_s_at	SLAM family member 8	SLAMF8	14.24	4.52	NM_020125
202948_at	interleukin 1 receptor, type I	IL1R1	5.67	3.31	NM_000877
209959_at	nuclear receptor subfamily 4, group A, member 3	NR4A3	5.20	2.48	U12767
211372_s_at	interleukin 1 receptor, type II	IL1R2	6.57	4.27	U64094
219890_at	C-type lectin domain family 5, member A	CLEC5A	16.91	5.64	NM_013252
202581_at	heat shock 70kDa protein 1A	HSPA1A	6.94	10.43	NM_005345
206697_s_at	haptoglobin	HP	-2.71	-2.34	NM_005143
203915_at	chemokine (C-X-C motif) ligand 9	CXCL9/MIG	11.16	8.48	NM_002416
209686_at	S100 calcium binding protein B	S100B	7.80	7.82	NM_006272
**immune response**					
209771_x_at	CD24 molecule	CD24	-8.30	-2.06	NM_013230
205789_at	CD1d molecule	CD1D	-4.49	-2.62	NM_001766
206277_at	purinergic receptor P2Y, G-protein coupled, 2	P2RY2	-2.90	-2.2	NM_002564
216984_x_at	immunoglobulin lambda variable 2–11	IGLV2-11	-3.66	-3.31	D84143
206983_at	chemokine (C-C motif) receptor 6	CCR6	2.01	2.67	NM_004367
205476_at	chemokine (C-C motif) ligand 20	CCL20	3.61	2.14	NM_004591
220307_at	CD244 molecule, natural killer cell receptor 2B4	CD244/2B4	-4.05	-2.73	AF242540
210029_at	indoleamine 2,3-dioxygenase 1	IDO1	5.87	2.34	M34455
205544_s_at	complement component receptor 2	CR2	-5.05	-3.12	NM_001877
220068_at	pre-B lymphocyte 3	VPREB3	-3.59	-3.43	NM_013378
207840_at	CD160 molecule	CD160	-14.75	-2.51	NM_007053
**apoptosis**					
217996_at	pleckstrin homology-like domain, family A, member 1	PHLDA1	6.09	4.32	NM_007350
204998_s_at	activating transcription factor 5	ATF5	5.79	2.79	NM_012068
204201_s_at	protein tyrosine phosphatase, non-receptor type 13	PTPN13	2.18	2.16	NM_006264
204614_at	serpin peptidase inhibitor, clade B, member 2	SERPINB2/PAI2	-5.90	-2.46	NM_002575
**cell cycle regulation**					
204115_at	guanine nucleotide binding protein (G protein), gamma 11	GNG11	-42.28	-2.65	NM_004126
**cell proliferation**					
201830_s_at	neuroepithelial cell transforming 1	NET1	4.27	3.05	NM_005863
201324_at	epithelial membrane protein 1	EMP1	45.31	2.06	NM_001423
208623_s_at	ezrin	EZR	2.41	3.04	J05021
209481_at	SNF related kinase	SNRK	-2.15	-2.54	AF226044
209815_at	patched homolog 1 (Drosophila)	PTCH1	-7.22	-2.65	NM_000264
209808_x_at	inhibitor of growth family, member 1	ING1	-2.08	-2.68	AF149723
202286_s_at	tumor-associated calcium signal transducer 2	TACSTD2	56.82	3.76	NM_002353
**ECM/ECM remodeling**					
210042_s_at	cathepsin Z	CTSZ	7.14	2.67	NM_001336
212158_at	syndecan 2	SDC2	15.68	2.84	NM_002998
202071_at	syndecan 4	SDC4	5.98	2.71	NM_002999
200665_s_at	secreted protein, acidic, cysteine-rich (osteonectin)	SPARC	-5.60	-2.11	NM_003118
206310_at	serine peptidase inhibitor, Kazal type 2	SPINK2	-2.30	-2.32	NM_021114
214768_x_at	anti-thyroid peroxidase monoclonal autoantibody IgK chain, V region	FAM20B	-4.03	-2.52	NM_014864
**angiogenesis**					
212070_at	G protein-coupled receptor 56	GPR56	-7.31	-3.25	NM_001145774
218723_s_at	chromosome 13 open reading frame 15	C13orf15	3.11	2.74	NM_014059
201110_s_at	thrombospondin 1	THBS1	-8.29	-3.48	NM_003246
**bone remodeling**					
206026_s_at	tumor necrosis factor, alpha-induced protein 6	TNFAIP6	4.84	4.23	NM_007115
202283_at	serpin peptidase inhibitor, clade F, member 1	SERPINF1	4.77	2.77	NM_002615
222258_s_at	SH3-domain binding protein 4	SH3BP4	3.73	3.11	AF015043
203395_s_at	hairy and enhancer of split 1	HES1	-2.18	-2.23	NM_005524
208328_s_at	myocyte enhancer factor 2A	MEF2A	2.51	2.45	NM_005587
212151_at	pre-B-cell leukemia homeobox 1	PBX1	-2.36	-2.2	NM_002585
205548_s_at	BTG family, member 3	BTG3/ANA	2.29	4.76	NM_006806
212531_at	lipocalin 2	LCN2	-3.26	-3.43	NM_005564
213222_at	phospholipase C, beta 1 (phosphoinositide-specific)	PLCB1	-3.68	-2.73	NM_182734
201417_at	SRY (sex determining region Y)-box 4	SOX4	-2.78	-3.03	NM_003107
210786_s_at	Friend leukemia virus integration 1	FLI1/ETS1	-2.25	-2.16	M93255
209875_s_at	secreted phosphoprotein 1	SPP1/OPN	448.78	4.32	J04765
201141_at	glycoprotein (transmembrane) nmb	GPNMB	147.53	3.27	NM_001005340
203411_s_at	lamin A/C	LMNA	13.75	2.4	NM_005572
202252_at	RAB13, member RAS oncogene family	RAB13	5.37	2.62	NM_002870
**signaling pathways**					
201020_at	tyrosine 3-monooxygenase	YWHAH	3.27	2.37	NM_003405
221211_s_at	chromosome 21 open reading frame 7	C21orf7	-8.00	-2.81	NM_020152
206020_at	suppressor of cytokine signaling 6	SOCS6	2.26	3.15	NM_004232
202581_at	heat shock 70kDa protein 1A	HSPA1A	6.94	10.43	NM_005345
209185_s_at	insulin receptor substrate 2	IRS2	-4.83	-2.05	AF073310
203395_s_at	hairy and enhancer of split 1	HES1	-2.18	-2.23	NM_005524
214453_s_at	interferon-induced protein 44	IFI44	2.32	2.06	NM_006417
204439_at	interferon-induced protein 44-like	IFI44L	2.23	2.34	NM_006820
204415_at	interferon, alpha-inducible protein 6	IFI6	2.20	3.04	NM_022873
213797_at	radical S-adenosyl methionine domain containing 2	RSAD2	3.68	2.65	NM_080657
203153_at	interferon-induced protein with tetratricopeptide repeats 1	IFIT1	3.17	2.21	NM_001548
204747_at	interferon-induced protein with tetratricopeptide repeats 3	IFIT3	2.58	3.06	NM_001549
205469_s_at	interferon regulatory factor 5	IRF5	2.03	2.78	NM_001098629
205483_s_at	ISG15 ubiquitin-like modifier	ISG15	2.03	2.23	NM_005101
202086_at	myxovirus resistance 1, interferon-inducible protein p78	MX1	3.05	2.66	NM_002462
205003_at	dedicator of cytokinesis 4	DOCK4	13.81	2.04	AY233380
216511_s_at	transcription factor 4	TCF7L2/TCF4	-7.27	-2.25	NM_001146274
**cell junctions**					
204627_s_at	integrin, beta 3	ITGB3	-9.21	-2.46	NM_000212
206493_at	integrin, alpha 2b	ITGA2B	-5.36	-2.33	NM_000419
**microtubule-based process**					
204141_at	tubulin, beta 2A	TUBB2A	5.21	3.06	NM_001069
**transport**					
209267_s_at	solute carrier family 39 (zinc transporter), member 8	SLC39A8	6.68	2.96	AB040120
209610_s_at	solute carrier family 1, member 4	SLC1A4	2.58	2.27	NM_003038
220974_x_at	sideroflexin 3	SFXN3	2.93	2.14	NM_030971
201243_s_at	ATPase, Na+/K+ transporting, beta 1 polypeptide	ATP1B1	3.86	2.97	NM_001677
**others**					
221541_at	cysteine-rich secretory protein LCCL domain containing 2	CRISPLD2	-6.35	-2.52	NM_031476
206643_at	histidine ammonia-lyase	HAL	-4.98	-3.37	NM_002108
209574_s_at	chromosome 18 open reading frame 1	C18orf1/LDLRAD4	2.27	2.13	NM_181481
203518_at	lysosomal trafficking regulator	LYST	-2.31	-2.17	NM_000081
208442_s_at	ataxia telangiectasia mutated	ATM	-3.32	-3.2	NM_000051
207156_at	histone cluster 1, H2ag	HIST1H2AG	-3.58	-2.04	NM_021064
204838_s_at	mutL homolog 3	MLH3	-4.14	-2.33	NM_014381
218711_s_at	serum deprivation response	SDPR	-19.65	-2.04	NM_004657
218865_at	MOCO sulphurase C-terminal domain containing 1	MOSC1/MARC1	-4.86	-2.45	NM_022746
213022_s_at	utrophin	UTRN/DRP1	-2.15	-2.59	NM_007124

### Frequency of IL-17–positive CD4+T cells in PBMCs from patients with PsA

Using flow cytometry, we evaluated the intracellular expression of the cytokine IL-17, by PBMCs from 30 patients with PsA and from 20 healthy control subjects, following stimulation with microbeads coated with anti-CD3 and CD28 or with Candida Albicans. We observed a higher proportion of IL-17–producing CD4+ T cells within the PBMCs of patients with PsA compared with healthy control subjects.

**Fig 3:**
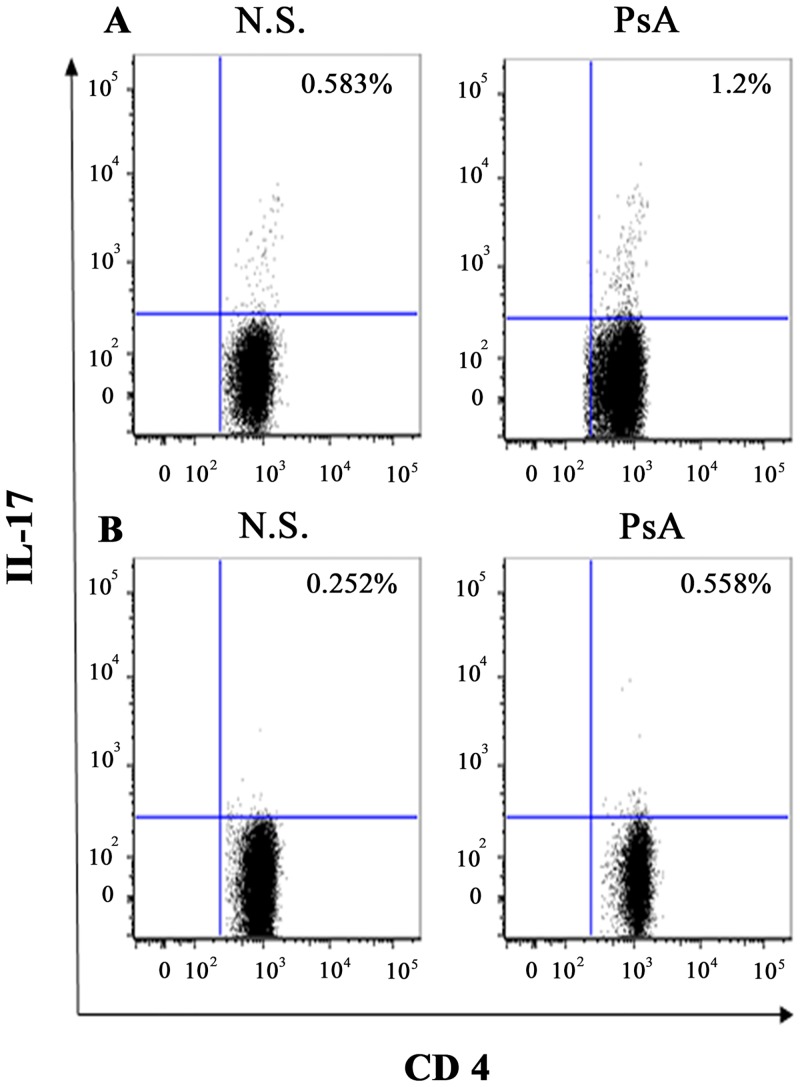
Flow cytometric analysis of IL-17 producing CD4+ lymphocytes in patients with PsA. Data are representative of all the 30 subjects studied. A and B panels show the percentages of IL-17 producing CD4+ lymphocytes in PBMC of healthy donors (N.S) and patients affected by PsA (PsA) after stimulation with Dynabeads Human T-Activator CD3/CD28 (panel a) or with heat-inactivated *Candida albicans* (panel b).

The mean values obtained in 30 PsA PBMC following stimulation with anti-CD3 and CD28 were 1.3% +/- 0.28 versus 0.65% +/- 0.2 (p = 0.005), while the mean values obtained in PSA PBMC following stimulation with Candida Albicans were 0.63% +/- 0.18 versus 0.33%+/- 0.07 (p = 0.004). Representative FACS experiments are shown in Fig (3A and 3B).

### Detection of IL-17 and IL-23 in synovial fluids from patients with PsA

Il-17 and IL-23 levels were investigated in 20 SFs of patients with PsA and in 20 SF of patients with OA used as controls. SF IL-17 levels were higher in PSA patients (17.87±11.13 pg/mL) than in controls (5.12±1.30 pg/mL) (*p*<0.01). SF IL-23 levels were higher in PSA patients (37.17±18.13 pg/mL) than in controls (14.12±7.23 pg/mL) (*p*<0.01)

### Detection of soluble mediators in PsA sera

**Fig 4:**
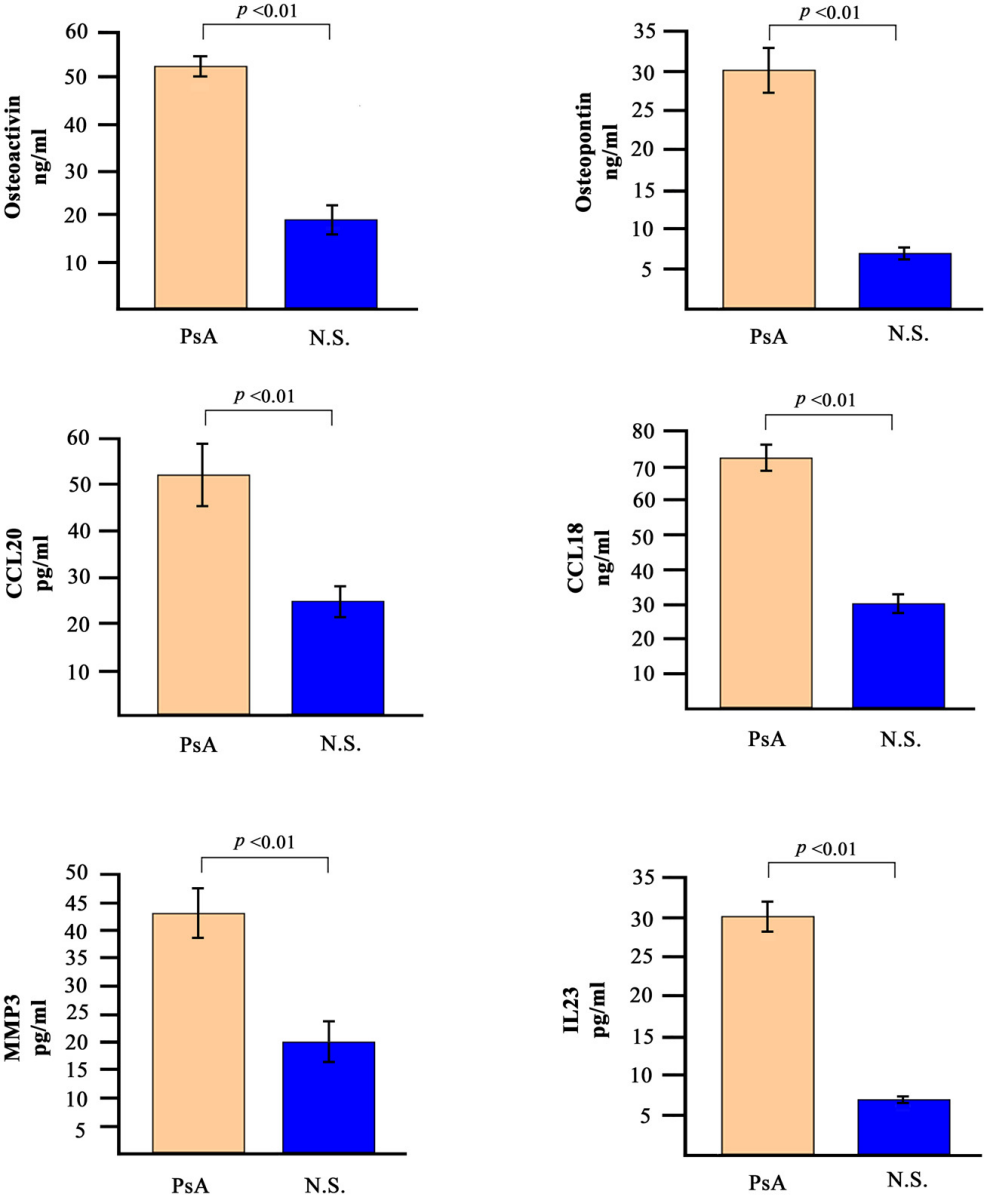
Serum levels of selected soluble mediators in PsA patients and in normal subjects. The histograms represent the mean of the results obtained in 30 healthy donors and in 30 PsA patients. *p* values were calculated using the non-parametric Mann-Whitney test.

The analysis of gene expression profiles was paralleled by the detection of some of the corresponding soluble mediators in the sera of patients with PsA. We decided to analyse the levels of osteopontin, osteoactivin, CCL20, CCL18, MMP-3 and IL-23. Fig 4 shows the concentration of these molecules in the sera of the 30 PsA patients. The serum levels of all the molecules tested was significantly higher in PsA patients when compared to 30 normal healthy donors.

**Fig 5:**
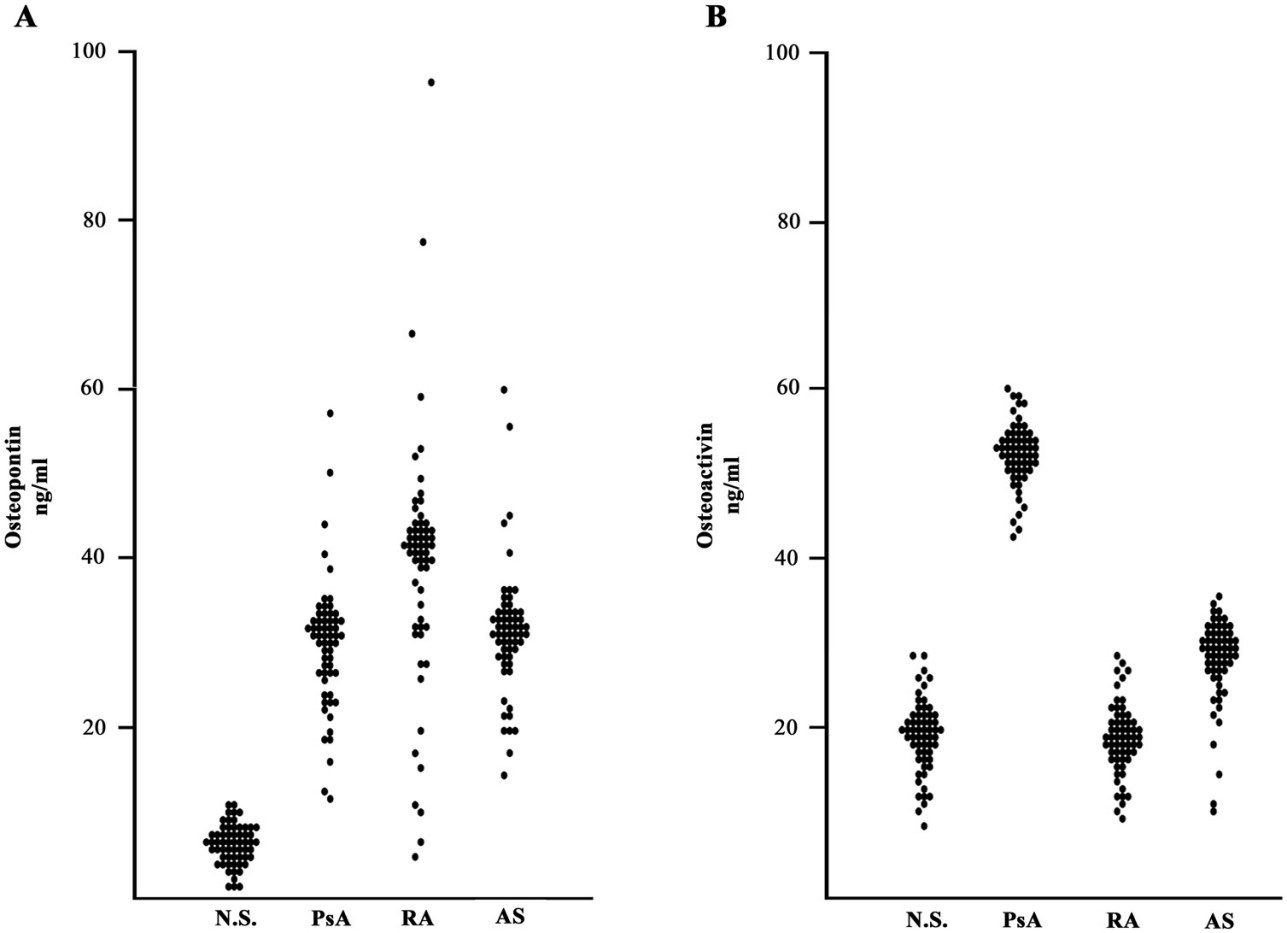
Serum levels of osteopontin and osteoactivin in PsA patients and in control subjects. Graphical representation of the distributions of osteopontin (A) and osteoactivin (B) serum levels in: 60 psoriatic arthritis (PsA) patients, 60 rheumatoid arthritis (RA) patients, 60 ankylosing spondylitis (AS) patients and in 60 normal subjects (NS). *p* values were calculated using the non-parametric Mann-Whitney test: Osteopontin: PsA vs NS: *p* <0.01; PsA vs SA: *p* <0.01, PsA vs AR: *p* not significant. Osteoactivin: PsA vs NS: *p* <0.01; PsA vs SA: *p* <0.05; PsA vs AR: *p* <0.01.

**Fig 6:**
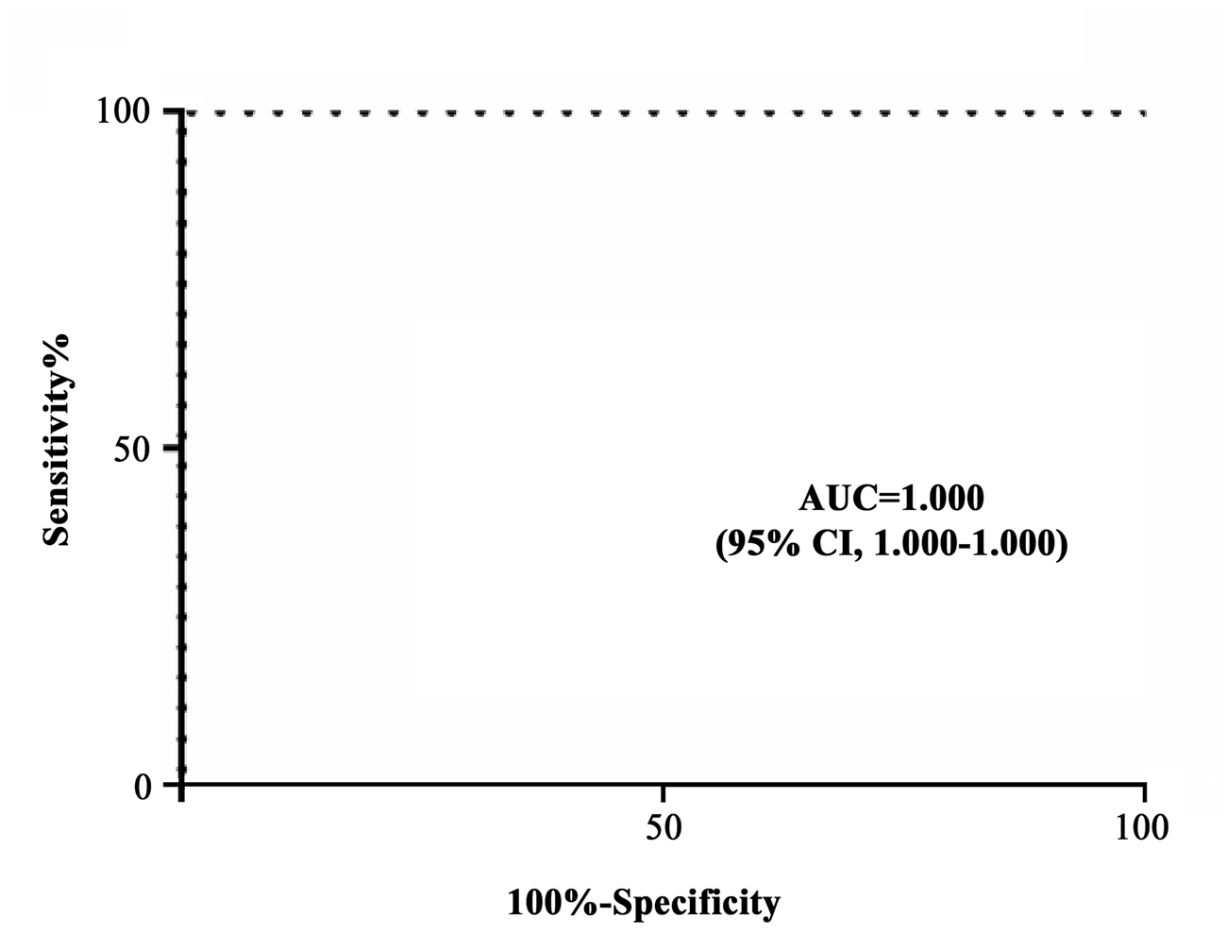
Sensitivity and specificity of the assay of osteoactivin levels between PsA patients and control subjects. AUC: area under the curve; CI: confidence interval.

When osteopontin and osteoactivin were evaluated in the entire cohort of 60 patients with PsA and compared with 60 normal subjects and with 60 patients affected by RA and AS, osteoactivin was found to be statistically higher in subjects with PsA (Fig 5) with a high sensitivity and specificity (Fig 6).

## Discussion

In this paper we provide for the first time a comprehensive analysis of the transcriptome within SM and PBC obtained from the same patients affected by PsA. We believe that the combined analysis of the gene expression profiles of paired synovial biopsies and PBC of patients with PsA is likely to better dissect the complex molecular pathways that regulate the different clinical and histopatological aspects of the disease. One contribution of this work is therefore a detailed compilation of genes relevant to the pathogenesis of PsA. Indeed the vast majority of DEGs is involved in biological processes closely connected to the main features of the disease. Noteworthy we observed that, when comparing DEGs in synovial biopsies with DEGs in PBC, a large number of genes were similarly modulated in the two settings, indicating that PBC may be a significant promise for gene expression studies as substitute of tissues that are not easily accessible.

A feature of PsA histopathology is the synovial membrane lining layer hyperplasia, which in RA has been at least in part attributed to an impairement of fibroblast-like-synoviocyte (FLS) apoptosis [120]. Our analysis of apoptosis-related genes indicate a predominant overexpression of anti-apoptotic transcripts which may contribute to the cell accumulation phenomena typical of synovial hyperplasia.

The synovial hyperplastic growth may also be sustained by the global upregulation of genes with mitogenic effect and by the repression of anti-proliferative transcripts observed in PsA samples.

Interestingly, the overexpression of the GAS6 and EZR genes has been already associated with FLS proliferation in inflammatory arthitides [60,121]. FLSs are indeed the predominant cell type in synovial membrane and, especially when switching their phenotype to macrophage-like cells, they can destroy extracellular matrix, deeply penetrating into bone and cartilage [122]. It is worthwhile mentioning that EZR has already been reported as differentially expressed in PsA by Pollock et al. [123]

In PsA synovium, genes involved in cell migration and tissue invasion are strongly upregulated (ie ETV5, and FABP5) consistently with the acquisition of a proinvasive phenotype by PsA synoviocytes. This behaviour may also depend on overexpression of extracellular matrix degrading enzymes.

In this regard we found a strong upregulation of transcripts encoding for proteases (i.e. MMP3, IDS, and CTSZ) and for the metalloproteases inducers ETV5 and FSCN1 [124], whereas proteinases inhibitors (i.e. PCSK5 and SPINK2) showed a decreased expression.

The upregulation of the MMP3 gene is particularly interesting, since its overexpression has been reported in PsA synovium [125] and increased MMP3 levels have been detected in sera of PsA patients by several investigators [126] including ourselves (Fig 4).

Among genes coding for ECM components, the upregulation of the two syndecans transcribing genes (SDC2 and SDC4) is remarkable since these molecules are involved in the retention and activation of leukocytes in inflamed synovium [127,128] and can induce synovial fibroblasts to produce cartilage matrix degrading enzymes such as ADAMTS5 [129].

Another aspect associated to synovial hyperplasia is neoangiogenesis, considered a typical feature of the early phase of PsA [130,131]. Synovial angiogenesis is mediated by several factors produced by both synovial tissue and infiltrating inflammatory cells [131] and promotes the synovial infiltration into the intraarticular cartilage [132,133].

Our gene array data show an overall up-regulation of proangiogenic genes involved in different steps of neoangiogenesis, such as GPC4, abundantly expressed in PsA synovium [75], and MAPK11, also known as p38β, a downstream target of VEGF signaling during angiogenesis [78]. An increased expression for MUC1, an activator of multiple pro-angiogenic factors during hypoxia-driven angiogenesis [134], typical of inflammatory joint diseases [135], was also observed.

Moreover we found a decreased expression of two anti-angiogenic genes, GPR56, a potent inhibitor of vessel formation [136] and THBS1, which inhibits vasculogenesis through the triggering of CD36 on endothelial cells, leading to apoptosis of endothelial cells [137]. Interestingly, using a rat model of osteoarthritis (OA) Hsie et al. [138] demonstrated that THBS1 gene transfer significantly reduced microvessel density and inflammation, thus controlling the progression of the disease.

A very peculiar aspect of PsA pathology is the presence of new bone deposition.

In this context our DEGs indicate that the process of bone formation may be due to genes which regulate osteoblast differentiation (LMNA, CAMTA1 and LCN2) [79,86,139] and proliferation (PBX1, RGS2 and SOX4) [84,85,87,140] or correlate with the bone-forming capacity of mesenchymal stem cells (MSCs) (CADM1). As a matter of fact the synovial membrane has been recently considered a reservoir of MSCs able to differentiate in osteoblasts and chondroblasts [141,142].

Moreover some upregulated genes have specific roles in particular aspects of bone development, such as bone matrix mineralization (ENPP1 and TUFT1) [82], bone morphogenesis (ALCAM) and endochondral ossification (SPRED2). Noteworthy endochondral ossification plays an important role in the pathogenesis of spondyloarthropathies, including PsA [143].

DEGs are therefore consistent with the new bone formation process typical of PsA, and mostly characterized by ankylosis, periostitis, and syndesmophytes [143].

PsA is characterized by bone erosions mediated by osteoclasts at the synovial-bone junction. It is well known that bone erosion is sustained by the osteoclast proteolytic activity and indeed a great increase of the osteoclast-specific marker ACP5 gene, was present in our arrays [90]. Accordingly, we also noticed a remarkable induction for the SH3BP4 gene (FC 3.73), a positive regulator of autophagy, which is a TNFα-dependent process thought to play an important role in joint destruction [144,145]. Two important mediators of bone remodeling typically associated with PsA are RANK and TNF both upregulated in PsA samples. RANK plays a pivotal role in osteoclastogenesis since its gene product is a macrophage-colony stimulating factor (M-CSF)-inducible molecule located on the osteoclast precursor surface that, upon engagement by its ligand RANKL, induces these cells to differentiate [146].

Interestingly the RANKL expression on osteoblasts and other effector cell is primarily regulated by TNF and by Annexin II [147], the product of the gene ANXA2, also upregulated in our PsA samples (see S2 Table).

TNF is involved in a number of autoimmune /inflammatory diseases and is one of the major proinflammatory factors in arthritis causing joint inflammation and cartilage destruction [148]. TNF level is increased both in the synovium and in the synovial fluid of PSA patients [149].

Besides these wellknown effects on inflammation and on bone resorption by inducing osteoclastogenesis and osteoclast recruitment, TNF is also able to induce the expression of the glycoprotein dickkopf-1 (DKK-1), the Wnt-signaling antagonist that suppresses the bone-formation process and the production of the osteoclastogenesis inhibitor, osteoprotegerin [92].

However several phase-3 studies showed that TNF inhibitors significantly ameliorated radiographic progression of the disease, but failed to control new bone formation [92]. It has been suggested that continued suppression of inflammation via anti-TNF agents may accelerate new bone formation and ankylosis [92] possibly through upregulation of DKK-1.

As expected, a large number of modulated genes encode for proinflammatory transcripts and the highest levels of induction were observed for FN1 (F.C. 405) followed by OLR1 (F.C. 83) and CXCL13 (F.C. 79).

Fibronectin is a component of the cartilage matrix but is also locally produced in the synovial fluid where its level positively correlates with joint destruction [150]. Moreover citrullinated forms of fibronectin (cFn) are commonly present in inflammed synovium [150] and notably, fibronectin is a target of autoantibodies in RA [151]. Furthermore cFn inhibits apoptosis and stimulates the secretion of proinflammatory cytokines in FLS isolated from RA patients [150] and induces osteoblast differentiation from human mesenchymal stem cells [152].

OLR1/LOX-1, the receptor for LDL oxidized expressed by endothelial cells and macrophages in the atherosclerotic lesions, is also produced by FLS in the RA synovium where it drives inflammation and cartilage degradation by inducing MMP-1 and MMP-3 expression [153].

CXCL13 is a B cell chemoattractant that has been related to the ectopic lymphoid neogenesis and T/B cells aggregates seen in PsA synovial tissues [130,154].

Other proinflammatory mediators highly induced in PsA samples include CXCL9/MIG, CCL18, CXCR6. CCL18 has been found increased in synovial tissue from RA patients [155] whereas MIG has been involved in regulating leucocyte traffic in both RA and PsA synovium [156].

CXCR6 is highly expressed by skin CD8^+^ T cells of psoriatic skin lesions [157] and in PsA synovium [158] where it has been suggested to play a role in T-cell homing [157].

We also found increased expression for the IL1R1 gene, an important regulator in both inflammation and autoimmunity. Indeed anti-IL-1 strategies have had a huge impact in autoimmune and inflammatory diseases [159].

Our results indicate a conspicuous participation of macrophages in the PSA synovium inflammatory infiltrate as showed by the up-regulation of several macrophage related genes, such as: FCGR2B, NR4A3, and CLEC5A/MDL1. Indeed macrophages are the principal source of TNFα [160] and in the inflammatory setting, TNF-α in synergy with RANKL, can induce the acquisition of the osteoclast phenotype by macrophages [161] thus contributing to promote osteolysis.

Among the above mentioned genes, NR4A3 and CLEC5A/MDL1 are abundantly expressed by activated macrophages [162,163]. Interestingly, during joint inflammation, overexpression of CLEC5A/MDL-1 recruits inflammatory cells and induces the production of IL-1, IL-6, IL-17A, and TNF, contributing to cartilage damage and bone erosion. FCGR2B, present on several cell types including synovial mononuclear phagocytes, monocytes, neutrophils and myeloid dendritic cells (DCs) [164] is an inhibitory receptor with important regulatory functions on Fc receptor activation, which has been found overexpressed in RA synovium samples [165]. Noteworthy variation in the gene encoding FCGR2B has been associated with susceptibility to autoimmune disease such as RA and SLE [166].

Another important gene cluster is the signal transduction gene category which includes several modulated transcripts particularly important for PsA pathogenesis. Indeed we noticed overexpression of 13 IFIG in PsA synovium, thus indicating the presence of an IFN type I signature, typically associated with autoimmune disease such as SLE, RA, Crohn’s disease and Sjogren syndrome [94–100]. Noteworthy a type I interferon signature is present also in DEGs from PBC of patients with PsA, indicating that indeed peripheral blood cells reflects, although at lower level, the gene modulation present in the synovium.

Finally, DEGs indicate a T cell immune response with prevalent upregulation of several Th17 related genes both in PsA synovium (8 upregulated transcripts) and, to a lesser extent, in PBC (5 upregulated genes). Th17 cells have been implicated in autoimmune diseases including SLE, RA and psoriasis. In these last two disease conditions, experimental evidence indicates that Th17 cells and the related IL-17/IL-23 cytokine axis, play a role in disease pathogenesis in animal models and in the human disease suggesting that targeting the IL-17/IL23 cytokine axis may represent a promising therapeutic strategy. In this regard it is now clear that Th17 cells and related cytokines play a crucial role in sustaining autoimmunity especially when associated to type I IFN-driven inflammation. A recent report [20] shows that CCR6^+^ memory T-helper cells producing IL-17A, IL-17F, IL-21, and/or IL-22 are increased in SLE patients and that this increase is related to the presence of IFN type I signature thus providing evidence that IFN type I signature co-acts with Th17 cells and related cytokines in the pathogenesis of systemic autoimmune diseases such as SLE.

In order to further confirm our gene expression data on overexpression of IFIG and Th17 pathways, we analysed the presence of IL-17 producing CD4+ T cells and found a significantly increased percentage of these cells in PBMC of patients with PsA compared with normal subjects. Moreover the levels of IL-17 and IL-23 in synovial fluid were higher than in control synovial fluids further confirming the findings of the gene array analysis. Taken together these data suggest an autoimmune origin of PsA, probably through the activation of the IL-23/IL-17 cytokine network. Indeed PsA has always been considered of autoimmune origin, driven by autoreactive T cells directed against autoantigens present in the skin and in the joints. This view has been recently questioned by McGonagle et al. [167] who have proposed that PsA may be considered an autoinflammatory rather than an autoimmune disease. One of the reasons adduced is that the autoimmune model would fail to explain diffuse enthesitis, and that the proposed autoantigens common to skin and joints have not been identified so far. However a recent work by Sherlock et al. [168,169]shows that IL-23 is able to induce enthesitis in animal models of spondyloarthropathy acting on resident T cells within the enthesis; once activated these entheseal T cells can promote local inflammation and bone remodeling through a variety of effector molecules such as IL-17 and IL-22. Therefore these findings suggest that mechanisms other then local injury (microtrauma, microdamage, altered vascularity and repair) [170] may be responsible for the onset of enthesitis and that enthesitis may be sustained by effector mediators of the Th17 cell subset. As far as the absence of autoantigens common to skin and joints adduced as evidence for an autoinflammatory origin of the disease we have recently found that PsA is characterized by the presence of serum autoantibodies crossreacting with an epitope shared by skin and joint antigens [171].

Finally our work shows that the two genes, SSP1 and GPNMB, encoding for osteopontin and osteoactivin are among the most expressed genes found in the gene array analysis (FC 450 and 147). Osteopontin (OPN), also called bone sialoprotein I (BSP-1 or BNSP), is encoded by the *SPP1* gene (secreted phosphoprotein 1), first identified in osteoblasts. OPN is biosynthesized by a variety of cells including fibroblasts, osteoblasts, osteocytes, chondrocytes, some bone marrow cells, dendritic cells, endothelial cells. OPN plays a role in bone metabolism (bone mineralization and remodeling) but has also a role in immunity. OPN is a cytokine with pleiotropic effect produced by activated T cells, dendritic cells and macrophages. It is released at increased levels during inflammation and the secreted form (s-OPN) has been shown to act as a chemoattractant for many cells through integrin receptors and CD44. Recently, it has been shown that OPN is able to regulate the expression of Toll like receptor-9 (TLR-9), of TLR-7-dependent interferon-alpha (IFN-alpha) in plasmacytoid dendritic cells. Moreover it seems to be implicated in Th17 developmenent [172]. Indeed OPN seems to play a role in the pathogenesis of several autoimmune diseases including RA, MS and SLE [173]. Osteoactivin (OA), also known as transmembrane glycoprotein GPNMB, is a type I glycoprotein expressed in osteoblasts, osteoclasts, melanocytes and other cell types. In osteoblast progenitors OA is a positive regulator of osteoblast differentiation in the final phases of matrix maturation and mineralization [174]. In addition, as observed in bone fracture in animal models, OA is able to accelerate bone repair [175].

We have found that osteopontin is elevated also in the sera of subjects with RA and AS, whereas high levels of osteoactivin are present only in the sera of PsA patients.

In conclusion, we report here the gene array analysis of paired SM and PBC in patients with PsA; the analysis has identified the modulation of cluster of genes encoding for molecules involved in the pathogenesis of the disease. The findings have been validated with different methods and further support that PsA is of autoimmune origin.

Moreover, one of the more expressed genes encodes for a molecule, osteoactivin, that is selectively elevated in the sera of patients with PsA suggesting that this molecule may be used as a marker of the disease.

## Supporting Information

S1 Table Annotated genes differentially expressed in PsA synovial membrane versus healthy synovial membrane grouped according to their function. (DOC)

S2 Table Annotated genes differentially expressed in PsA PBC versus healthy controls grouped according to their function. (DOC)
