# Supplementary material for: Gene Expression Profiling in Peripheral Blood Cells and Synovial Membranes of Patients with Psoriatic Arthritis
Source: PLoS One. 2015 Jun 18;10(6):e0128262. doi: 10.1371/journal.pone.0128262 (PMC4473102; doi:10.1371/journal.pone.0128262)
Supplement: S1 Table — (DOC) [file pone.0128262.s001.doc]

| **Table S1. Annotated genes differentially expressed in PsA synovial membrane versus healthy synovial membrane grouped according to their function** | | | | |
| --- | --- | --- | --- | --- |
| **Probe Set ID** | **Gene Title** | **Gene Symbol** | **FC** | **Representative Public ID** |
| **angiogenesis** |  |  |  |  |
| 204984_at | glypican 4 | GPC4 | 2.24 | NM_001448 |
| 201110_s_at | thrombospondin 1 | THBS1 | -8.29 | NM_003246 |
| 212070_at | G protein-coupled receptor 56 | GPR56 | -7.31 | NM_001145774 |
| 219501_at | ecto-NOX disulfide-thiol exchanger 1 | ENOX1 | 3.47 | NM_017993 |
| 219278_at | mitogen-activated protein kinase kinase kinase 6 | MAP3K6 | 2.41 | NM_004672 |
| 208961_s_at | Kruppel-like factor 6 | KLF6 | 2.25 | AB017493 |
| 211499_s_at | mitogen-activated protein kinase 11 | MAPK11 | 2.24 | NM_002751 |
| 218723_s_at | chromosome 13 open reading frame 15 | C13orf15 | 3.11 | NM_014059 |
| 213693_s_at | mucin 1, cell surface associated | MUC1 | 2.46 | X80761 |
| **apoptosis** |  |  |  |  |
| 205573_s_at | sorting nexin 7 | SNX7 | 7.76 | NM_015976 |
| 217996_at | pleckstrin homology-like domain, family A, member 1 | PHLDA1 | 6.09 | NM_007350 |
| 204998_s_at | activating transcription factor 5 | ATF5 | 5.79 | NM_012068 |
| 221530_s_at | basic helix-loop-helix family, member e41 | BHLHE41 | 5.03 | AB044088 |
| 217966_s_at | family with sequence similarity 129, member A | FAM129A | 3.54 | AF288391 |
| 211367_s_at | caspase 1, apoptosis-related cysteine peptidase | CASP1 | -2.83 | NM_033292 |
| 220048_at | ectodysplasin A receptor | EDAR | -2.22 | NM_022336 |
| 204201_s_at | protein tyrosine phosphatase, non-receptor type 13 | PTPN13 | 2.18 | NM_006264 |
| 243_g_at | microtubule-associated protein 4 | MAP4 | 2.40 | M64571 |
| 204614_at | serpin peptidase inhibitor, clade B, member 2 | SERPINB2/PAI2 | -5.90 | NM_002575 |
| **bone remodeling** | |  |  |  |
| 207113_s_at | tumor necrosis factor (TNF superfamily, member 2) | TNF | 3.55 | NM_000594 |
| 209875_s_at | secreted phosphoprotein 1 | SPP1/OPN | 448.78 | J04765 |
| 201141_at | glycoprotein (transmembrane) nmb | GPNMB | 147.53 | NM_001005340 |
| 206026_s_at | tumor necrosis factor, alpha-induced protein 6 | TNFAIP6 | 4.84 | NM_007115 |
| 202283_at | serpin peptidase inhibitor, clade F, member 1 | SERPINF1 | 4.77 | NM_002615 |
| 222258_s_at | SH3-domain binding protein 4 | SH3BP4 | 3.73 | AF015043 |
| 203395_s_at | hairy and enhancer of split 1 | HES1 | -2.18 | NM_005524 |
| 213503_x_at | annexin A2 | ANXA2 | 2.09 | NM_001002858 |
| 218872_at | tescalcin | TESC | -3.35 | NM_017899 |
| 211974_x_at | recombination signal binding protein for immunoglobulin kappa J region | RBPJ | 2.92 | NM_203284 |
| 201951_at | activated leukocyte cell adhesion molecule | ALCAM | 5.95 | L38608 |
| 213268_at | calmodulin binding transcription activator 1 | CAMTA1 | 2.80 | NM_001242701 |
| 208328_s_at | myocyte enhancer factor 2A | MEF2A | 2.51 | NM_005587 |
| 212151_at | pre-B-cell leukemia homeobox 1 | PBX1 | -2.36 | NM_002585 |
| 209031_at | cell adhesion molecule 1 | CADM1 | 14.85 | NM_014333 |
| 203411_s_at | lamin A/C | LMNA | 13.75 | NM_005572 |
| 202252_at | RAB13, member RAS oncogene family | RAB13 | 5.37 | NM_002870 |
| 202388_at | regulator of G-protein signaling 2, 24kDa | RGS2 | -4.34 | NM_002923 |
| 212458_at | sprouty-related, EVH1 domain containing 2 | SPRED2 | 4.33 | AY299090 |
| 204638_at | acid phosphatase 5, tartrate resistant | ACP5 | 3.91 | NM_001611 |
| 207037_at | tumor necrosis factor receptor superfamily, member 11a, NFKB activator | TNFRSF11A/RANK | 3.77 | NM_003839 |
| 205548_s_at | BTG family, member 3 | BTG3/ANA | 2.29 | NM_006806 |
| 205066_s_at | ectonucleotide pyrophosphatase/phosphodiesterase 1 | ENPP1 | 7.20 | NM_006208 |
| 212531_at | lipocalin 2 | LCN2 | -3.26 | NM_005564 |
| 213222_at | phospholipase C, beta 1 (phosphoinositide-specific) | PLCB1 | -3.68 | NM_182734 |
| 201417_at | SRY (sex determining region Y)-box 4 | SOX4 | -2.78 | NM_003107 |
| 210786_s_at | Friend leukemia virus integration 1 | FLI1/ETS1 | -2.25 | M93255 |
| 205807_s_at | tuftelin 1 | TUFT1 | 2.32 | NM_020127 |
| **cell junctions** |  |  |  |  |
| 201667_at | gap junction protein, alpha 1, 43kDa | GJA1 | 9.03 | NM_000165 |
| 207324_s_at | desmocollin 1 | DSC1 | -2.23 | NM_004948 |
| 204627_s_at | integrin, beta 3 | ITGB3 | -9.21 | NM_000212 |
| 206493_at | integrin, alpha 2b | ITGA2B | -5.36 | NM_000419 |
| **cell cycle regulation** | |  |  |  |
| 208712_at | cyclin D1 | CCND1 | 9.94 | NM_053056.2 |
| 220658_s_at | aryl hydrocarbon receptor nuclear translocator-like 2 | ARNTL2 | 4.03 | NM_020183 |
| 204115_at | guanine nucleotide binding protein (G protein), gamma 11 | GNG11 | -42.28 | NM_004126 |
| 204032_at | breast cancer anti-estrogen resistance 3 | BCAR3 | 3.94 | NM_003567 |
| **cell proliferation** | |  |  |  |
| 213348_at | cyclin-dependent kinase inhibitor 1C (p57, Kip2) | CDKN1C | -17.49 | NM_001122631 |
| 201830_s_at | neuroepithelial cell transforming 1 | NET1 | 4.27 | NM_005863 |
| 209815_at | patched homolog 1 (Drosophila) | PTCH1 | -7.22 | NM_000264 |
| 202177_at | growth arrest-specific 6 | GAS6 | 4.12 | NM_000820 |
| 205081_at | cysteine-rich protein 1 | CRIP1 | 3.11 | NM_001311 |
| 219787_s_at | epithelial cell transforming sequence 2 oncogene | ECT2 | 3.04 | NM_018098 |
| 209053_s_at | Wolf-Hirschhorn syndrome candidate 1 | WHSC1 | 2.26 | NM_133331 |
| 206074_s_at | high mobility group AT-hook 1 | HMGA1 | 2.01 | NM_002131 |
| 201324_at | epithelial membrane protein 1 | EMP1 | 45.31 | NM_001423 |
| 209481_at | SNF related kinase | SNRK | -2.15 | AF226044 |
| 209808_x_at | inhibitor of growth family, member 1 | ING1 | -2.08 | AF149723 |
| 208623_s_at | ezrin | EZR | 2.41 | J05021 |
| 202286_s_at | tumor-associated calcium signal transducer 2 | TACSTD2 | 56.82 | NM_002353 |
| **cell migration and/or invasion** | |  |  |  |
| 204040_at | ring finger protein 144A | RNF144A | -5.04 | NM_014746 |
| 217226_s_at | Human homeobox protein (PHOX1) | PHOX1/PRX1 | 3.16 | M95929 |
| 201564_s_at | fascin homolog 1, actin-bundling protein | FSCN1 | 3.16 | NM_003088 |
| 200650_s_at | lactate dehydrogenase A | LDHA | 2.31 | NM_005566 |
| 202345_s_at | fatty acid binding protein 5 (psoriasis-associated) | FABP5 | 12.73 | NM_001444 |
| 206298_at | Rho GTPase activating protein 22 | ARHGAP22 | 3.89 | NM_021226 |
| 218451_at | CUB domain containing protein 1 | CDCP1 | 3.61 | NM_022842 |
| 205805_s_at | receptor tyrosine kinase-like orphan receptor 1 | ROR1 | 2.02 | NM_005012 |
| 203349_s_at | ets variant 5 | ETV5 | 7.03 | NM_004454 |
| 200696_s_at | gelsolin (amyloidosis, Finnish type) | GSN | 3.65 | NM_000177 |
| **ECM/ECM remodeling** | |  |  |  |
| 205828_at | matrix metallopeptidase 3 (stromelysin 1, progelatinase) | MMP3 | 26.05 | NM_002422 |
| 217757_at | alpha-2-macroglobulin | A2M | 25.43 | NM_000014 |
| 210042_s_at | cathepsin Z | CTSZ | 7.14 | NM_001336 |
| 212158_at | syndecan 2 | SDC2 | 15.68 | NM_002998 |
| 202439_s_at | iduronate 2-sulfatase | IDS | 2.31 | NM_000202 |
| 202071_at | syndecan 4 | SDC4 | 5.98 | NM_002999 |
| 200665_s_at | secreted protein, acidic, cysteine-rich (osteonectin) | SPARC | -5.60 | NM_003118 |
| 205559_s_at | proprotein convertase subtilisin/kexin type 5 | PCSK5 | -4.52 | NM_006200 |
| 203184_at | fibrillin 2 | FBN2 | -4.59 | NM_001999 |
| 216905_s_at | suppression of tumorigenicity 14 | ST14 | 2.78 | NM_021978 |
| 206310_at | serine peptidase inhibitor, Kazal type 2 | SPINK2 | -2.30 | NM_021114 |
| 214768_x_at | anti-thyroid peroxidase monoclonal autoantibody IgK chain, V region | FAM20B | -4.03 | NM_014864 |
| **signaling pathways** | |  |  |  |
| 201020_at | tyrosine 3-monooxygenase/tryptophan 5-monooxygenase activation protein, eta | YWHAH | 3.27 | NM_003405 |
| 209050_s_at | ral guanine nucleotide dissociation stimulator | RALGDS | 2.43 | NM_006266 |
| 221211_s_at | chromosome 21 open reading frame 7 | C21orf7 | -8.00 | NM_020152 |
| 205003_at | dedicator of cytokinesis 4 | DOCK4 | 13.81 | AY233380 |
| 213693_s_at | mucin 1, cell surface associated | MUC1 | 2.46 | X80761 |
| 219889_at | frequently rearranged in advanced T-cell lymphomas | FRAT1 | -2.59 | NM_005479 |
| 206020_at | suppressor of cytokine signaling 6 | SOCS6 | 2.26 | NM_004232 |
| 216511_s_at | transcription factor 4 | TCF7L2/TCF4 | -7.27 | NM_001146274 |
| 219278_at | mitogen-activated protein kinase kinase kinase 6 | MAP3K6 | 2.41 | NM_004672 |
| 211499_s_at | mitogen-activated protein kinase 11 | MAPK11 | 2.24 | NM_002751 |
| 212912_at | ribosomal protein S6 kinase, 90kDa, polypeptide 2 | RPS6KA2 | 2.65 | NM_021135 |
| 202581_at | heat shock 70kDa protein 1A | HSPA1A | 6.94 | NM_005345 |
| 205805_s_at | receptor tyrosine kinase-like orphan receptor 1 | ROR1 | 2.02 | NM_005012 |
| 208712_at | cyclin D1 | CCND1 | 9.94 | NM_053056.2 |
| 201667_at | gap junction protein, alpha 1, 43kDa | GJA1 | 9.03 | NM_000165 |
| 209185_s_at | insulin receptor substrate 2 | IRS2 | -4.83 | AF073310 |
| 205066_s_at | ectonucleotide pyrophosphatase/phosphodiesterase 1 | ENPP1 | 7.20 | NM_006208 |
| 211974_x_at | recombination signal binding protein for immunoglobulin kappa J region | RBPJ | 2.92 | NM_203284 |
| 203395_s_at | hairy and enhancer of split 1 | HES1 | -2.18 | NM_005524 |
| 207113_s_at | tumor necrosis factor (TNF superfamily, member 2) | TNF | 3.55 | NM_000594 |
| 202411_at | interferon, alpha-inducible protein 27 | IFI27 | 13.74 | NM_005532 |
| 204439_at | interferon-induced protein 44-like | IFI44L | 2.23 | NM_006820 |
| 214453_s_at | interferon-induced protein 44 | IFI44 | 2.32 | NM_006417 |
| 204415_at | interferon, alpha-inducible protein 6 | IFI6 | 2.20 | NM_022873 |
| 213797_at | radical S-adenosyl methionine domain containing 2 | RSAD2 | 3.68 | NM_080657 |
| 217502_at | interferon-induced protein with tetratricopeptide repeats 2 | IFIT2 | 2.03 | NM_001547 |
| 203153_at | interferon-induced protein with tetratricopeptide repeats 1 | IFIT1 | 3.17 | NM_001548 |
| 204747_at | interferon-induced protein with tetratricopeptide repeats 3 | IFIT3 | 2.58 | NM_001549 |
| 219209_at | interferon induced with helicase C domain 1 | IFIH1 | 2.15 | NM_022168 |
| 205469_s_at | interferon regulatory factor 5 | IRF5 | 2.03 | NM_001098629 |
| 205483_s_at | ISG15 ubiquitin-like modifier | ISG15 | 2.03 | NM_005101 |
| 202086_at | myxovirus resistance 1, interferon-inducible protein p78 | MX1 | 3.05 | NM_002462 |
| 208436_s_at | interferon regulatory factor 7 | IRF7 | 2.15 | NM_001572 |
| **immune response** | |  |  |  |
| 205569_at | lysosomal-associated membrane protein 3 | LAMP3 | 36.99 | NM_014398 |
| 207111_at | egf-like module containing, mucin-like, hormone receptor-like 1 | EMR1 | -4.10 | NM_001974 |
| 204787_at | V-set and immunoglobulin domain containing 4 | VSIG4/Z39IG | 26.76 | NM_007268 |
| 201212_at | legumain | LGMN | 22.77 | NM_001008530 |
| 207840_at | CD160 molecule | CD160 | -14.75 | NM_007053 |
| 205267_at | POU class 2 associating factor 1 | POU2AF1/BOB1 | -10.69 | NM_006235 |
| 215784_at | CD1e molecule | CD1E | 9.41 | NM_030893 |
| 206991_s_at | chemokine (C-C motif) receptor 5 | CCR5 | 8.42 | NM_000579 |
| 209771_x_at | CD24 molecule | CD24 | -8.30 | NM_013230 |
| 210029_at | indoleamine 2,3-dioxygenase 1 | IDO1 | 5.87 | M34455 |
| 205544_s_at | complement component receptor 2 | CR2 | -5.05 | NM_001877 |
| 205789_at | CD1d molecule | CD1D | -4.49 | NM_001766 |
| 220307_at | CD244 molecule, natural killer cell receptor 2B4 | CD244/2B4 | -4.05 | AF242540 |
| 221239_s_at | Fc receptor-like 2 | FCRL2 | -3.90 | NM_030764 |
| 220068_at | pre-B lymphocyte 3 | VPREB3 | -3.59 | NM_013378 |
| 221538_s_at | plexin A1 | PLXNA1 | 3.36 | NM_032242 |
| 205297_s_at | CD79b molecule, immunoglobulin-associated beta | CD79B | -3.23 | NM_000626 |
| 214435_x_at | v-ral simian leukemia viral oncogene homolog A (ras related) | RALA | 3.11 | NM_005402 |
| 206453_s_at | NDRG family member 2 | NDRG2 | 3.10 | NM_016250 |
| 206277_at | purinergic receptor P2Y, G-protein coupled, 2 | P2RY2 | -2.90 | NM_002564 |
| 35974_at | lymphoid-restricted membrane protein | LRMP | -2.47 | NM_006152 |
| 208488_s_at | complement component (3b/4b) receptor 1 | CR1 | -2.36 | NM_000651 |
| 206682_at | C-type lectin domain family 10, member A | CLEC10A/MGL | 4.01 | NM_006344 |
| 216984_x_at | immunoglobulin lambda variable 2-11 | IGLV2-11 | -3.66 | D84143 |
| 216876_s_at | interleukin 17A | IL17A | 2.30 | U32659 |
| 221165_s_at | interleukin 22 | IL22 | 2.04 | AF279437 |
| 221111_at | interleukin 26 | IL26 | 3.67 | NM_018402 |
| 220054_at | interleukin 23, alpha subunit p19 | IL23A | 2.00 | NM_016584 |
| 208991_at | signal transducer and activator of transcription 3 (acute-phase response factor) | STAT3 | 2.26 | NM_139276.2 |
| 206983_at | chemokine (C-C motif) receptor 6 | CCR6 | 2.01 | NM_004367 |
| 205476_at | chemokine (C-C motif) ligand 20 | CCL20 | 3.61 | NM_004591 |
| **inflammation** |  |  |  |  |
| 207113_s_at | tumor necrosis factor (TNF superfamily, member 2) | TNF | 3.55 | NM_000594 |
| 210004_at | oxidized low density lipoprotein (lectin-like) receptor 1 | OLR1 | 83.10 | AF035776 |
| 205242_at | chemokine (C-X-C motif) ligand 13 | CXCL13 | 79.22 | NM_006419 |
| 32128_at | chemokine (C-C motif) ligand 18 | CCL18 | 20.32 | NM_002988 |
| 219386_s_at | SLAM family member 8 | SLAMF8 | 14.24 | NM_020125 |
| 214560_at | formyl peptide receptor 3 | FPR3 | 13.78 | NM_002030 |
| 203915_at | chemokine (C-X-C motif) ligand 9 | CXCL9/MIG | 11.16 | NM_002416 |
| 209686_at | S100 calcium binding protein B | S100B | 7.80 | NM_006272 |
| 200872_at | S100 calcium binding protein A10 | S100A10 | 2.21 | NM_002966 |
| 205479_s_at | plasminogen activator, urokinase | PLAU | 7.78 | NM_002658 |
| 209392_at | ectonucleotide pyrophosphatase/phosphodiesterase 2 | ENPP2/ATX | 6.33 | L35594 |
| 206974_at | chemokine (C-X-C motif) receptor 6 | CXCR6 | 6.28 | NM_006564 |
| 202948_at | interleukin 1 receptor, type I | IL1R1 | 5.67 | NM_000877 |
| 201841_s_at | heat shock 27kDa protein 1 | HSPB1 | 5.30 | NM_001540 |
| 210889_s_at | Fc fragment of IgG, low affinity IIb, receptor (CD32) | FCGR2B | 5.25 | M31933 |
| 209959_at | nuclear receptor subfamily 4, group A, member 3 | NR4A3 | 5.20 | U12767 |
| 215537_x_at | dimethylarginine dimethylaminohydrolase 2 | DDAH2 | 3.69 | NM_013974 |
| 218424_s_at | STEAP family member 3 | STEAP3 | 3.19 | NM_018234 |
| 205404_at | hydroxysteroid (11-beta) dehydrogenase 1 | HSD11B1 | 2.60 | NM_005525 |
| 208002_s_at | acyl-CoA thioesterase 7 | ACOT7 | 2.55 | NM_007274 |
| 209619_at | CD74 molecule, MHC, class II invariant chain | CD74 | 2.12 | NM_001025159 |
| 202686_s_at | AXL receptor tyrosine kinase | AXL | 21.22 | NM_021913 |
| 211719_x_at | fibronectin 1 | FN1 | 405.39 | NM_212482 |
| 211372_s_at | interleukin 1 receptor, type II | IL1R2 | 6.57 | U64094 |
| 206697_s_at | haptoglobin | HP | -2.71 | NM_005143 |
| 219890_at | C-type lectin domain family 5, member A | CLEC5A | 16.91 | NM_013252 |
| 202581_at | heat shock 70kDa protein 1A | HSPA1A | 6.94 | NM_005345 |
| 212296_at | proteasome 26S subunit, non-ATPase, 14 | PSMD14 | 2.07 | NM_005805 |
| 206295_at | interleukin 18 (interferon-gamma-inducing factor) | IL18 | 2.01 | NM_001562 |
| **transport** |  |  |  |  |
| 209267_s_at | solute carrier family 39 (zinc transporter), member 8 | SLC39A8 | 6.68 | AB040120 |
| 220974_x_at | sideroflexin 3 | SFXN3 | 2.93 | NM_030971 |
| 219911_s_at | solute carrier organic anion transporter family, member 4A1 | SLCO4A1 | 5.69 | NM_016354 |
| 219714_s_at | calcium channel, voltage-dependent, alpha 2/delta subunit 3 | CACNA2D3 | -5.26 | NM_018398 |
| 209610_s_at | solute carrier family 1, member 4 | SLC1A4 | 2.58 | NM_003038 |
| 201243_s_at | ATPase, Na+/K+ transporting, beta 1 polypeptide | ATP1B1 | 3.86 | NM_001677 |
| **microtubule-based process** | |  |  |  |
| 204141_at | tubulin, beta 2A | TUBB2A | 5.21 | NM_001069 |
| 213476_x_at | tubulin, beta 3 | TUBB3 | 2.75 | NM_006086 |
| 209191_at | tubulin, beta 6 | TUBB6 | 5.94 | NM_032525 |
| **others** |  |  |  |  |
| 206643_at | histidine ammonia-lyase | HAL | -4.98 | NM_002108 |
| 209574_s_at | chromosome 18 open reading frame 1 | C18orf1/LDLRAD4 | 2.27 | NM_181481 |
| 209005_at | F-box and leucine-rich repeat protein 5 | FBXL5 | -2.24 | NM_012161 |
| 203518_at | lysosomal trafficking regulator | LYST | -2.31 | NM_000081 |
| 208442_s_at | ataxia telangiectasia mutated | ATM | -3.32 | NM_000051 |
| 207156_at | histone cluster 1, H2ag | HIST1H2AG | -3.58 | NM_021064 |
| 204838_s_at | mutL homolog 3 | MLH3 | -4.14 | NM_014381 |
| 218711_s_at | serum deprivation response | SDPR | -19.65 | NM_004657 |
| 205632_s_at | phosphatidylinositol-4-phosphate 5-kinase, type I, beta | PIP5K1B | -4.26 | NM_003558 |
| 205709_s_at | CDP-diacylglycerol synthase 1 | CDS1 | 3.91 | U65887 |
| 218865_at | MOCO sulphurase C-terminal domain containing 1 | MOSC1/MARC1 | -4.86 | NM_022746 |
| 212360_at | adenosine monophosphate deaminase 2 | AMPD2 | -2.27 | NM_004037 |
| 207601_at | sulfotransferase family, cytosolic, 1B, member 1 | SULT1B1 | -2.27 | NM_014465 |
| 221541_at | cysteine-rich secretory protein LCCL domain containing 2 | CRISPLD2 | -6.35 | NM_031476 |
| 209829_at | family with sequence similarity 65, member B | FAM65B | -3.14 | NM_014722 |
| 213022_s_at | utrophin | UTRN/DRP1 | -2.15 | NM_007124 |
