# Supplementary material for: Gene Expression Profiling in Peripheral Blood Cells and Synovial Membranes of Patients with Psoriatic Arthritis
Source: PLoS One. 2015 Jun 18;10(6):e0128262. doi: 10.1371/journal.pone.0128262 (PMC4473102; doi:10.1371/journal.pone.0128262)
Supplement: S2 Table — (DOC) [file pone.0128262.s002.doc]

| **Table S2. Annotated genes differentially expressed in PsA PBC versus healthy controls grouped according to their function** | | | | |
| --- | --- | --- | --- | --- |
| **Probe Set ID** | **Gene title** | **Gene symbol** | **FC** | **Representative Public ID** |
| **Inflammation** |  |  |  |  |
| 211506_s_at | interleukin 8 | IL8 | 3.78 | AF043337 |
| 32128_at | chemokine (C-C motif) ligand 18 | CCL18 | 2.03 | NM_002988 |
| 204470_at | chemokine (C-X-C motif) ligand 1 | CXCL1 | 7.24 | NM_001511 |
| 204655_at | chemokine (C-C motif) ligand 5 | CCL5 | 2.55 | NM_002985 |
| 205128_x_at | prostaglandin-endoperoxide synthase 1 | PTGS1 | 2.67 | NM_000962 |
| 207850_at | chemokine (C-X-C motif) ligand 3 | CXCL3 | 3.12 | NM_002090 |
| 208200_at | interleukin 1, alpha | IL1A | 3.29 | NM_000575 |
| 209201_x_at | chemokine (C-X-C motif) receptor 4 | CXCR4 | 2.46 | AY242129 |
| 209774_x_at | chemokine (C-X-C motif) ligand 2 | CXCL2 | 3.67 | NM_002089 |
| 210004_at | oxidized low density lipoprotein (lectin-like) receptor 1 | OLR1 | 7.16 | AF035776 |
| 219386_s_at | SLAM family member 8 | SLAMF8 | 4.52 | NM_020125 |
| 202948_at | interleukin 1 receptor, type I | IL1R1 | 3.31 | NM_000877 |
| 209959_at | nuclear receptor subfamily 4, group A, member 3 | NR4A3 | 2.48 | U12767 |
| 211372_s_at | interleukin 1 receptor, type II | IL1R2 | 4.27 | U64094 |
| 206697_s_at | haptoglobin | HP | -2.34 | NM_005143 |
| 219890_at | C-type lectin domain family 5, member A | CLEC5A | 5.64 | NM_013252 |
| 202581_at | heat shock 70kDa protein 1A | HSPA1A | 10.43 | NM_005345 |
| 209901_x_at | allograft inflammatory factor 1 | AIF1 | 2.18 | U19713 |
| 209276_s_at | glutaredoxin (thioltransferase) | GLRX | -2.54 | NM_001243659 |
| 200824_at | glutathione S-transferase pi 1 | GSTP1 | -2.63 | NM_000852 |
| 212819_at | ankyrin repeat and SOCS box-containing 1 | ASB1 | -2.57 | NM_001040445 |
| 203915_at | chemokine (C-X-C motif) ligand 9 | CXCL9 | 8.48 | NM_002416 |
| 204720_s_at | DnaJ (Hsp40) homolog, subfamily C, member 6 | DNAJC6 | 3.66 | NM_001256864 |
| 209686_at | S100 calcium binding protein B | S100B | 7.82 | NM_006272 |
| 214953_s_at | amyloid beta (A4) precursor protein | APP | 2.06 | X06989 |
| **Immune response** | |  |  |  |
| 205798_at | interleukin 7 receptor | IL7R | 2.56 | NM_002185 |
| 206341_at | interleukin 2 receptor, alpha | IL2RA | 2.27 | NM_000417 |
| 206693_at | interleukin 7 | IL7 | 3.45 | NM_000880 |
| 209771_x_at | CD24 molecule | CD24 | -2.06 | NM_013230 |
| 210029_at | indoleamine 2,3-dioxygenase 1 | IDO1 | 2.34 | M34455 |
| 205544_s_at | complement component (3d/Epstein Barr virus) receptor 2 | CR2 | -3.12 | NM_001877 |
| 205789_at | CD1d molecule | CD1D | -2.62 | NM_001766 |
| 220307_at | CD244 molecule, natural killer cell receptor 2B4 | CD244 | -2.73 | AF242540 |
| 220068_at | pre-B lymphocyte 3 | VPREB3 | -3.43 | NM_013378 |
| 206277_at | purinergic receptor P2Y, G-protein coupled, 2 | P2RY2 | -2.20 | NM_002564 |
| 216984_x_at | immunoglobulin lambda variable 2-11 | IGLV2-11 | -3.31 | D84143 |
| 206983_at | chemokine (C-C motif) receptor 6 | CCR6 | 2.67 | NM_004367 |
| 205476_at | chemokine (C-C motif) ligand 20 | CCL20 | 2.14 | NM_004591 |
| 206828_at | TXK tyrosine kinase | TXK | -2.82 | NM_003328 |
| 200041_s_at | HLA-B associated transcript 1 | BAT1 | 2.49 | NM_004640 |
| 200917_s_at | signal recognition particle receptor (docking protein) | SRPR | 3.62 | NM_003139 |
| 221417_x_at | sphingosine-1-phosphate receptor 5 | S1PR5 | 2.10 | NM_030760 |
| 205099_s_at | chemokine (C-C motif) receptor 1 | CCR1 | 2.68 | NM_001295 |
| 207840_at | CD160 molecule | CD160 | -2.51 | NM_007053 |
| 211597_s_at | HOP homeobox | HOPX | -2.19 | NM_032495 |
| 210354_at | interferon, gamma | IFNG | -2.94 | X01992 |
| 215967_s_at | lymphocyte antigen 9 | LY9 | 2.54 | NM_002348 |
| 208930_s_at | interleukin enhancer binding factor 3, 90kDa | ILF3 | 2.53 | NM_012218 |
| 214470_at | killer cell lectin-like receptor subfamily B, member 1 | KLRB1 | -2.37 | NM_002258 |
| 215339_at | natural killer-tumor recognition sequence | NKTR | 2.28 | NM_005385 |
| 202516_s_at | discs, large homolog 1 | DLG1 | -2.50 | NM_004087 |
| 203263_s_at | Cdc42 guanine nucleotide exchange factor (GEF) 9 | ARHGEF9 | -2.52 | NM_015185 |
| 206999_at | interleukin 12 receptor, beta | IL12RB | 2.62 | NM_005535 |
| 210313_at | leukocyte immunoglobulin-like receptor, subfamily A, member 4 | LILRA4 | -2.48 | NM_012276 |
| 215633_x_at | leukocyte specific transcript 1 | LST1 | -2.74 | NM_007161 |
| 204718_at | EPH receptor B6 | EPHB6 | 2.47 | NM_004445 |
| 204863_s_at | interleukin 6 signal transducer (gp130, oncostatin M receptor) | IL6ST | 3.87 | AB102799 |
| 204506_at | protein phosphatase 3 , regulatory subunit B, alpha isoform | PPP3R1 | 2.01 | NM_000945 |
| 201005_at | CD9 molecule | CD9 | 2.76 | NM_001769 |
| 212420_at | E74-like factor 1 (ets domain transcription factor) | ELF1 | 2.60 | NM_001145353 |
| 221058_s_at | chemokine-like factor | CKLF | -2.53 | NM_016326 |
| **Apoptosis** |  |  |  |  |
| 201846_s_at | RING1 and YY1 binding protein | RYBP | 2.03 | NM_012234 |
| 217996_at | pleckstrin homology-like domain, family A, member 1 | PHLDA1 | 4.32 | NM_007350 |
| 204998_s_at | activating transcription factor 5 | ATF5 | 2.79 | NM_012068 |
| 204201_s_at | protein tyrosine phosphatase, non-receptor type 13 | PTPN13 | 2.16 | NM_006264 |
| 216836_s_at | v-erb-b2 erythroblastic leukemia viral oncogene homolog 2 | ERBB2 | -2.60 | X03363 |
| 219423_x_at | tumor necrosis factor receptor superfamily, member 25 | TNFRSF25 | 2.04 | NM_003790 |
| 205554_s_at | deoxyribonuclease I-like 3 | DNASE1L3 | -2.26 | NM_004944 |
| 209526_s_at | hepatoma-derived growth factor, related protein 3 | HDGFRP3 | 3.23 | AB029156 |
| 212106_at | Fas associated factor family member 2 | FAF2 | 2.06 | NM_014613 |
| 201251_at | pyruvate kinase, muscle | PKM2 | 2.16 | NM_002654 |
| 204614_at | serpin peptidase inhibitor, clade B (ovalbumin), member 2 | SERPINB2 | -2.46 | NM_002575 |
| **Cell cycle regulation** | |  |  |  |
| 203376_at | cell division cycle 40 homolog (S. cerevisiae) | CDC40 | 2.46 | NM_015891 |
| 218555_at | anaphase promoting complex subunit 2 | ANAPC2 | 3.25 | NM_013366 |
| 220072_at | centrosome and spindle pole associated protein 1 | CSPP1 | 2.57 | NM_024790 |
| 204115_at | guanine nucleotide binding protein (G protein), gamma 11 | GNG11 | -2.65 | NM_004126 |
| 201702_s_at | protein phosphatase 1, regulatory (inhibitor) subunit 10 | PPP1R10 | 2.79 | NM_002714 |
| **Cell proliferation** | |  |  |  |
| 201830_s_at | neuroepithelial cell transforming 1 | NET1 | 3.05 | NM_005863 |
| 201324_at | epithelial membrane protein 1 | EMP1 | 2.06 | NM_001423 |
| 209481_at | SNF related kinase | SNRK | -2.54 | AF226044 |
| 202286_s_at | tumor-associated calcium signal transducer 2 | TACSTD2 | 3.76 | NM_002353 |
| 201897_s_at | CDC28 protein kinase regulatory subunit 1B | CKS1B | -2.06 | NM_001826 |
| 208623_s_at | ezrin | EZR | 3.04 | J05021 |
| 209808_x_at | inhibitor of growth family, member 1 | ING1 | -2.68 | AF149723 |
| 210428_s_at | hepatocyte growth factor-regulated tyrosine kinase substrate | HGS | 3.47 | AF260566 |
| 209815_at | patched homolog 1 | PTCH1 | -2.65 | NM_000264 |
| 203210_s_at | replication factor C (activator 1) 5, 36.5kDa | RFC5 | -2.51 | NM_007370 |
| **ECM/ECM remodeling** | |  |  |  |
| 222073_at | collagen, type IV, alpha 3 (Goodpasture antigen) | COL4A3 | 2.03 | NM_000091 |
| 219403_s_at | heparanase | HPSE | 2.52 | AF155510 |
| 203936_s_at | matrix metallopeptidase 9 | MMP9 | 2.72 | NM_004994 |
| 204475_at | matrix metallopeptidase 1 | MMP1 | 2.04 | NM_002421 |
| 210042_s_at | cathepsin Z | CTSZ | 2.67 | NM_001336 |
| 212158_at | syndecan 2 | SDC2 | 2.84 | NM_002998 |
| 202071_at | syndecan 4 | SDC4 | 2.71 | NM_002999 |
| 200665_s_at | secreted protein, acidic, cysteine-rich (osteonectin) | SPARC | -2.11 | NM_003118 |
| 206310_at | serine peptidase inhibitor, Kazal type 2 | SPINK2 | -2.32 | NM_021114 |
| 214768_x_at | anti-thyroid peroxidase monoclonal autoantibody IgK chain, V region | FAM20B | -2.52 | NM_014864 |
| 211343_s_at | collagen, type XIII, alpha 1 | COL13A1 | -2.43 | NM_080801 |
| 201666_at | TIMP metallopeptidase inhibitor 1 | TIMP1 | -3.02 | NM_003254 |
| 214895_s_at | ADAM metallopeptidase domain 10 | ADAM10 | -2.81 | AF009615 |
| **Angiogenesis** |  |  |  |  |
| 212070_at | G protein-coupled receptor 56 | GPR56 | -3.25 | NM_001145774 |
| 218723_s_at | chromosome 13 open reading frame 15 | C13orf15 | 2.74 | NM_014059 |
| 201110_s_at |  | THBS1 | -3.48 | NM_003246 |
| 209946_at | vascular endothelial growth factor C | VEGFC | 3.57 | NM_005429 |
| **Bone remodeling** | |  |  |  |
| 206026_s_at | tumor necrosis factor, alpha-induced protein 6 | TNFAIP6 | 4.23 | NM_007115 |
| 202283_at | serpin peptidase inhibitor, clade F, member 1 | SERPINF1 | 2.77 | NM_002615 |
| 222258_s_at | SH3-domain binding protein 4 | SH3BP4 | 3.11 | AF015043 |
| 203395_s_at | hairy and enhancer of split 1 | HES1 | -2.23 | NM_005524 |
| 208328_s_at | myocyte enhancer factor 2A | MEF2A | 2.45 | NM_005587 |
| 212151_at | pre-B-cell leukemia homeobox 1 | PBX1 | -2.20 | NM_002585 |
| 205548_s_at | BTG family, member 3 | BTG3 | 4.76 | NM_006806 |
| 212531_at | lipocalin 2 | LCN2 | -3.43 | NM_005564 |
| 213222_at | phospholipase C, beta 1 | PLCB1 | -2.73 | NM_182734 |
| 201417_at | SRY (sex determining region Y)-box 4 | SOX4 | -3.03 | NM_003107 |
| 210786_s_at | Friend leukemia virus integration 1 | FLI1 | -2.16 | M93255 |
| 209875_s_at | secreted phosphoprotein 1 | SPP1 | 4.32 | J04765 |
| 201141_at | glycoprotein (transmembrane) nmb | GPNMB | 3.27 | NM_001005340 |
| 203411_s_at | lamin A/C | LMNA | 2.40 | NM_005572 |
| 202252_at | RAB13, member RAS oncogene family | RAB13 | 2.62 | NM_002870 |
| 200771_at | laminin, gamma 1 (formerly LAMB2) | LAMC1 | 2.21 | NM_002293 |
| 203276_at | lamin B1 | LMNB1 | 2.18 | NM_005573 |
| **Signal transduction** | |  |  |  |
| 201020_at | tyrosine 3-monooxygenase | YWHAH | 2.37 | NM_003405 |
| 221211_s_at | chromosome 21 open reading frame 7 | C21orf7 | -2.81 | NM_020152 |
| 206020_at | suppressor of cytokine signaling 6 | SOCS6 | 3.15 | NM_004232 |
| 202581_at | heat shock 70kDa protein 1A | HSPA1A | 10.43 | NM_005345 |
| 209185_s_at | insulin receptor substrate 2 | IRS2 | -2.05 | AF073310 |
| 203395_s_at | hairy and enhancer of split 1, (Drosophila) | HES1 | -2.23 | NM_005524 |
| 204439_at | interferon-induced protein 44-like | IFI44L | 2.34 | NM_006820 |
| 214453_s_at | CDNA FLJ56519 complete cds, highly similar to Interferon-induced protein 44 | IFI44 | 2.06 | NM_006417 |
| 204415_at | interferon, alpha-inducible protein 6 | IFI6 | 3.04 | NM_022873 |
| 213797_at | radical S-adenosyl methionine domain containing 2 | RSAD2 | 2.65 | NM_080657 |
| 203153_at | interferon-induced protein with tetratricopeptide repeats 1 | IFIT1 | 2.21 | NM_001548 |
| 204747_at | interferon-induced protein with tetratricopeptide repeats 3 | IFIT3 | 3.06 | NM_001549 |
| 205469_s_at | interferon regulatory factor 5 | IRF5 | 2.78 | NM_001098629 |
| 205483_s_at | ISG15 ubiquitin-like modifier | ISG15 | 2.23 | NM_005101 |
| 202086_at | myxovirus resistance 1, interferon-inducible protein p78 | MX1 | 2.66 | NM_002462 |
| 205003_at | dedicator of cytokinesis 4 | DOCK4 | 2.04 | AY233380 |
| 216511_s_at | transcription factor 7-like 2 | TCF7L2 | -2.25 | NM_001146274 |
| 208965_s_at | interferon, gamma-inducible protein 16 | IFI16 | 2.56 | NM_001206567 |
| 216206_x_at | mitogen-activated protein kinase kinase 7 | MAP2K7 | 2.66 | NM_145185 |
| 215188_at | serine/threonine kinase 24 | STK24 | 2.10 | NM_003576 |
| 208820_at | PTK2 protein tyrosine kinase 2 | PTK2 | 2.76 | NM_153831 |
| 209896_s_at | protein tyrosine phosphatase, non-receptor type 11 | PTPN11 | -2.36 | NM_002834 |
| 201996_s_at | spen homolog, transcriptional regulator | SPEN | 2.98 | NM_015001 |
| 201868_s_at | transducin (beta)-like 1X-linked | TBL1X | 2.00 | NM_005647 |
| 203304_at | BMP and activin membrane-bound inhibitor homolog | BAMBI | 3.50 | NM_012342 |
| **Cell junction** |  |  |  |  |
| 205884_at | integrin, alpha 4 | ITGA4 | -2.29 | NM_000885 |
| 205055_at | integrin, alpha E | ITGAE | -2.01 | NM_002208 |
| 212036_s_at | pinin, desmosome associated protein | PNN | -2.47 | NM_002687 |
| 204627_s_at | integrin, beta 3 | ITGB3 | -2.46 | NM_000212 |
| 206493_at | integrin, alpha 2b | ITGA2B | -2.33 | NM_000419 |
| **Metabolism** |  |  |  |  |
| 206177_s_at | arginase, liver | ARG1 | 2.72 | NM_000045 |
| 210069_at | choline kinase beta | CHKB | 2.30 | NM_005198 |
| 203032_s_at | fumarate hydratase | FH | -2.20 | NM_000143 |
| 210154_at | malic enzyme 2, NAD(+)-dependent, mitochondrial | ME2 | -2.94 | NM_002396 |
| 215069_at | N-myristoyltransferase 2 | NMT2 | 2.17 | NM_004808 |
| 202839_s_at | NADH dehydrogenase (ubiquinone) 1 beta subcomplex, 7, 18kDa | NDUFB7 | -2.27 | NM_004146 |
| 218357_s_at | translocase of inner mitochondrial membrane 8 homolog B (yeast) | TIMM8B | -2.37 | NM_012459 |
| **microtubule based process** | |  |  |  |
| 204141_at | tubulin, beta 2A | TUBB2A | 3.06 | NM_001069 |
| **Transport** |  |  |  |  |
| 214769_at | chloride channel 4 | CLCN4 | 2.94 | NM_001830 |
| 201999_s_at | dynein, light chain, Tctex-type 1 | DYNLT1 | -2.48 | NM_006519 |
| 207237_at | potassium voltage-gated channel, shaker-related subfamily, member 3 | KCNA3 | 2.07 | NM_002232 |
| 204401_at | potassium calcium-activated channel, subfamily N, member 4 | KCNN4 | 2.61 | NM_002250 |
| 202083_s_at | SEC14-like 1 | SEC14L1 | 3.44 | NM_003003 |
| 221920_s_at | solute carrier family 25, member 37 | SLC25A37 | 2.14 | NM_016612 |
| 209267_s_at | solute carrier family 39 (zinc transporter), member 8 | SLC39A8 | 2.96 | AB040120 |
| 220974_x_at | sideroflexin 3 | SFXN3 | 2.14 | NM_030971 |
| 209610_s_at | solute carrier family 1, member 4 | SLC1A4 | 2.27 | NM_003038 |
| 201243_s_at | ATPase, Na+/K+ transporting, beta 1 polypeptide | ATP1B1 | 2.97 | NM_001677 |
| 208751_at | N-ethylmaleimide-sensitive factor attachment protein, alpha | NAPA | 2.52 | NM_003827 |
| 202203_s_at | autocrine motility factor receptor | AMFR | 2.28 | NM_001144 |
| 201879_at | ariadne homolog, ubiquitin-conjugating enzyme E2 binding protein, 1 (Drosophila) | ARIH1 | 2.57 | NM_005744 |
| 217826_s_at | ubiquitin-conjugating enzyme E2, J1 | UBE2J1 | 3.51 | NM_016021 |
| 201523_x_at | ubiquitin-conjugating enzyme E2N | UBE2N | -2.49 | NM_003348 |
| 220370_s_at | ubiquitin specific peptidase 36 | USP36 | 2.75 | NM_025090 |
| **Others** |  |  |  |  |
| 213022_s_at | utrophin | UTRN | -2.59 | NM_007124 |
| 221541_at | cysteine-rich secretory protein LCCL domain containing 2 | CRISPLD2 | -2.52 | NM_031476 |
| 206643_at | histidine ammonia-lyase | HAL | -3.37 | NM_002108 |
| 209574_s_at | chromosome 18 open reading frame 1 | C18orf1 | 2.13 | NM_181481 |
| 203518_at | lysosomal trafficking regulator | LYST | -2.17 | NM_000081 |
| 207156_at | histone cluster 1, H2ag | HIST1H2AG | -2.04 | NM_021064 |
| 204838_s_at | mutL homolog 3 | MLH3 | -2.33 | NM_014381 |
| 218711_s_at | serum deprivation response (phosphatidylserine binding protein) | SDPR | -2.04 | NM_004657 |
| 218865_at | MOCO sulphurase C-terminal domain containing 1 | MOSC1 | -2.45 | NM_022746 |
| 208442_s_at | ataxia telangiectasia mutated | ATM | -3.20 | NM_000051 |
| 35436_at | golgi autoantigen, golgin subfamily a, 2 | GOLGA2 | 2.61 | NM_004486 |
| 208797_s_at | golgi autoantigen, golgin subfamily a, 8A | GOLGA8A | 2.29 | AF204231 |
| 204929_s_at | vesicle-associated membrane protein 5 | VAMP5 | -2.56 | NM_006634 |
| 220221_at | vacuolar protein sorting 13 homolog D | VPS13D | 2.80 | NM_018156 |
|  |  |  |  |  |
